# Cost-effectiveness of metabolic surgery for the treatment of type 2 diabetes and obesity: a systematic review of economic evaluations

**DOI:** 10.1007/s10198-022-01494-2

**Published:** 2022-07-22

**Authors:** Karen Jordan, Christopher G. Fawsitt, Paul G. Carty, Barbara Clyne, Conor Teljeur, Patricia Harrington, Mairin Ryan

**Affiliations:** 1 RCSI University of Medicine and Health Sciences, Dublin, Ireland; 2 Health Information and Quality Authority, Dublin, Ireland; 3 Department of General Practice, Royal College of Surgeons in Ireland, Dublin, Ireland; 4 Department of Pharmacology and Therapeutics, Trinity College Dublin, Trinity Health Sciences, St James’s Hospital, Dublin 8, Ireland

**Keywords:** Diabetes, Metabolic surgery, Bariatric surgery, Systematic review, Cost-effectiveness, Economic evaluation, I1 Health, I18 Government policy, Public health

## Abstract

**Aim:**

To systematically identify and appraise the international literature on the cost-effectiveness of metabolic surgery for the treatment of comorbid type 2 diabetes (T2D) and obesity.

**Methods:**

A systematic search was conducted in electronic databases and grey literature sources up to 20 January 2021. Economic evaluations in a T2D population or a subpopulation with T2D were eligible for inclusion. Screening, data extraction, critical appraisal of methodological quality (Consensus Health Economic Criteria list) and assessment of transferability (International Society for Pharmacoeconomics and Outcomes Research questionnaire) were undertaken in duplicate. The incremental cost-effectiveness ratio (ICER) was the main outcome. Costs were reported in 2020 Irish Euro. Cost-effectiveness was interpreted using willingness-to-pay (WTP) thresholds of €20,000 and €45,000/quality-adjusted life year (QALY). Due to heterogeneity arising from various sources, a narrative synthesis was undertaken.

**Results:**

Thirty studies across seventeen jurisdictions met the inclusion criteria; 16 specifically in a T2D population and 14 in a subpopulation with T2D. Overall, metabolic surgery was found to be cost-effective or cost-saving. Where undertaken, the results were robust to sensitivity and scenario analyses. Of the 30 studies included, 15 were considered high quality. Identified limitations included limited long-term follow-up data and uncertainty regarding the utility associated with T2D remission.

**Conclusion:**

Published high-quality studies suggest metabolic surgery is a cost-effective or cost-saving intervention. As the prevalence of obesity and obesity-related diseases increases worldwide, significant investment and careful consideration of the resource requirements needed for metabolic surgery programmes will be necessary to ensure that service provision is adequate to meet demand.

**Supplementary Information:**

The online version contains supplementary material available at 10.1007/s10198-022-01494-2.

## Introduction

The prevalence of type 2 diabetes (T2D) is considerably higher in Western Europe than the global average and continues to rise despite preventive measures [1]. T2D causes serious microvascular (e.g. retinopathy, nephropathy and neuropathy) and macrovascular complications (e.g. stroke and myocardial infarction), leading to considerable morbidity and mortality [2]. As a result, T2D imposes an economic burden on individuals, society and healthcare systems [3, 4]. It has been estimated that the burden of diabetes on health systems and economies will continue to rise based on current trends. The cost of diabetes will represent an estimated 1.9% of global gross domestic product (GDP) by 2030 [5]. Therefore, interventions that improve the management of T2D have the potential to yield considerable clinical and economic benefits.

Best medical care for T2D may include behaviour change, pharmacological treatment and self-management support [6–9]. However, for patients whose treatment targets are not being met despite best medical care, treatment options are limited. Metabolic surgery has been recommended by professional organisations including the American Diabetes Association (ADA) and the International Federation for the Surgery of Obesity and Metabolic Diseases (IFSO) to improve glycaemic control and reduce cardiovascular risk factors in patients with T2D and obesity, leading to the coining of the term “metabolic surgery” [8, 10]. Metabolic surgery refers to the use of bariatric surgery procedures, such as Roux-en-Y gastric bypass (RYGB) or sleeve gastrectomy (SG), with the aim of improving T2D control in patients with comorbid T2D and obesity. Despite substantial research activity to support its use, metabolic surgery remains an underutilised treatment option for patients with comorbid T2D and obesity potentially due to high upfront costs and misconceptions surrounding its use solely as a weight-loss intervention [11–14].

Previous systematic reviews have synthesised the evidence of the cost-effectiveness of bariatric surgery as a weight-loss intervention in the population with obesity [15–17]. However, these results are not generalisable to the population with comorbid T2D and obesity as the potential costs and outcomes of disease management differ considerably when compared with the overall population with obesity [18]. The aims of this systematic review were to: (1) synthesise and critically appraise the available evidence on the cost-effectiveness of metabolic surgery compared with usual care and (2) identify factors that influence the cost-effectiveness of metabolic surgery in patients with comorbid T2D and obesity.

## Methods

This systematic review is reported according to the Preferred Reporting Items for Systematic Reviews and Meta-Analyses (PRISMA) criteria and consistent with recently published guidance on systematic reviews with cost and cost-effectiveness outcomes [19, 20]. The review was prospectively registered on PROSPERO (CRD42021234932).

### Search strategy

The review question was developed in accordance with the population, intervention, comparator, outcomes, study design (PICOS) framework. Studies were considered eligible for inclusion if they reported the cost-effectiveness of metabolic surgery for the treatment of adults with comorbid T2D and obesity. Full details of the inclusion criteria are provided in the Supplementary Information, Table S1.

Electronic searches were carried out in Medline (via Ovid) and Embase on 20 January 2021 and were supplemented with a grey literature search of international health technology assessment (HTA) repositories. The electronic search strategy was developed in consultation with an information specialist. Clinical search terms were combined with the Scottish Intercollegiate Guidelines Network (SIGN) economic search filters [21]. The reference lists of included studies were searched to identify additional relevant studies. No date limits were applied to the search (Supplementary Information, Tables S2 and S3).

### Study identification, data extraction and critical appraisal

Two reviewers independently screened titles and abstracts in Covidence®. The full texts of potentially eligible studies were retrieved and independently assessed by two reviewers. Study characteristics and results were extracted by two reviewers using a standardised, pre-piloted electronic data extraction form. The main outcome was the incremental cost-effectiveness ratio (ICER), typically expressed in terms of the cost per quality-adjusted life year (QALY) gained. For cost-effectiveness analyses (CEA), where QALYs were not used as the measure of effect, other outcomes (for example, cost per case of diabetes remitted) were extracted. Factors that may influence the cost-effectiveness of surgery, identified through subgroup analysis, were also extracted. The methodological quality of included economic evaluations was assessed using the Consensus on Health Economics Criteria (CHEC)-list [22]. The International Society for Pharmacoeconomics (ISPOR) questionnaire was used to determine the transferability of model-based economic evaluations [23]. Disagreements regarding study eligibility, data extraction and critical appraisal were resolved through discussion, or if necessary, a third reviewer. Critical appraisal plots (CHEC-list and ISPOR) and cost-effectiveness planes were produced in Excel 2013.

### Data synthesis

In line with ISPOR best practice recommendations, the cost-effectiveness of model-based (parameters are based on multiple sources) and empirical evidence-based (parameters are based on a single study such as a randomised controlled trial) economic evaluations were synthesised separately [20]. Due to heterogeneity arising from various sources, a narrative synthesis was undertaken.

There is no best practice method for synthesis of economic evidence, which depends on the purpose of the review [20]. This systematic review was undertaken to inform decision-making in Ireland regarding the potential introduction of a metabolic surgery programme into the publicly funded health system. Accordingly, the main analysis was undertaken from the Irish perspective. ICERs were typically reported as the country-specific currency at a specific point in time. To facilitate comparison of ICERs across countries and years, where appropriate, costs were transformed to a common year and currency (2020 Euro) using consumer price indices (CPI) and purchasing power parities (PPP) (i.e. adjusted ICERs) [24]. The selected PPP reference was Euro costs in Ireland. WTP thresholds of €20,000 and €45,000 per QALY gained, commonly employed in Ireland and consistent with empirically based thresholds in other high income countries, were adopted as reference points for guiding interpretation of cost-effectiveness [24–26]. Unadjusted ICERs as reported by included studies and context-specific WTP threshold are presented in the Supplementary Information, Table S6.

For studies where only subgroup-specific ICERs were presented, a weighted average ICER for the overall population of interest was calculated based on the population characteristics provided in the original study or correspondence with study authors. For studies where information on the study population characteristics was not available, a simple average was calculated.

## Results

### Search results

**Fig. 1:**
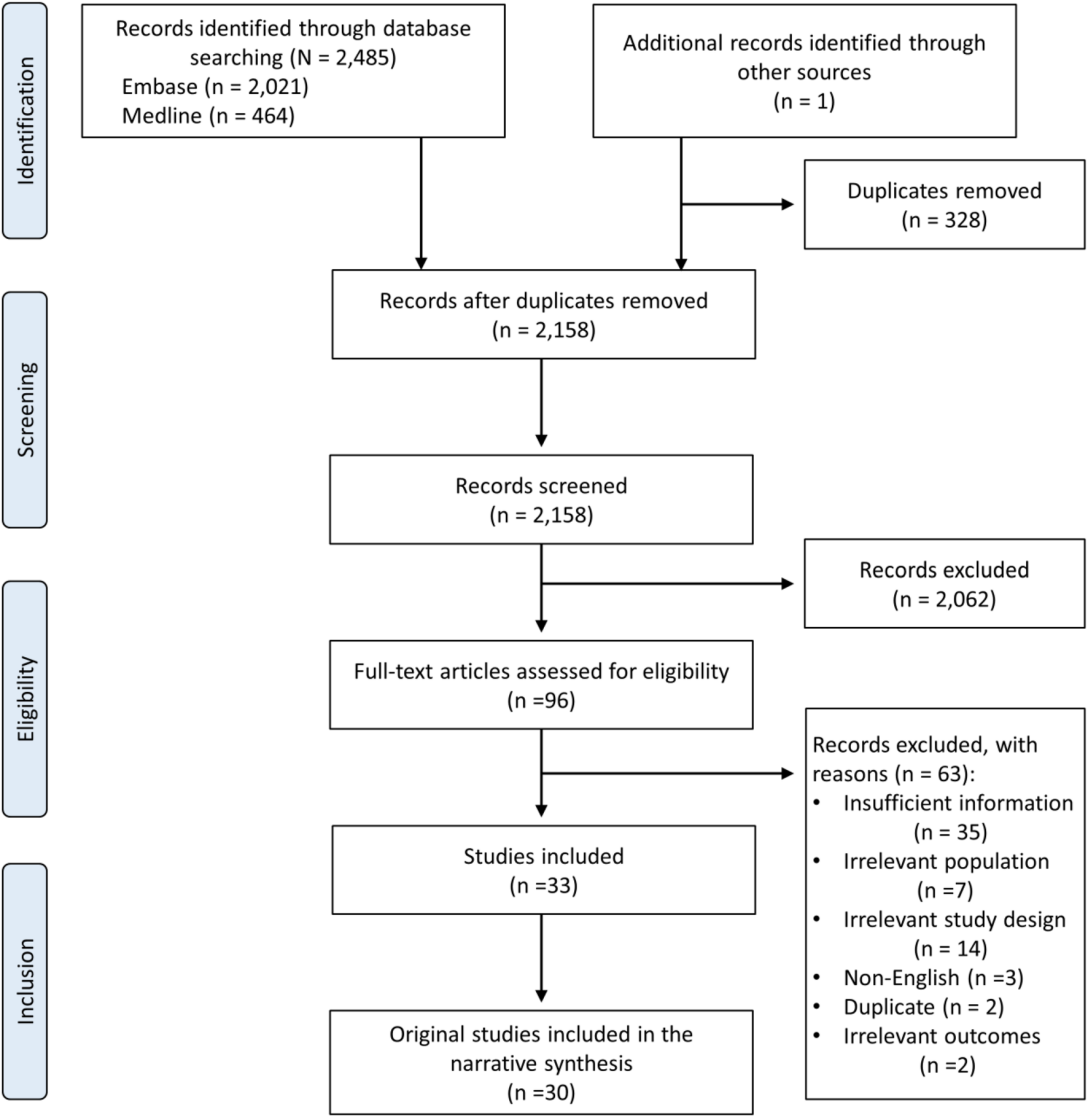
PRISMA flow diagram of search and selection process

After removal of duplicate articles, 2,158 titles and abstracts were assessed for eligibility. Ninety-six articles required full‐text review. Thirty original articles from 33 publications fulfilled the inclusion criteria. Of these, 16 reported on the cost-effectiveness of metabolic surgery specifically in a T2D population [27–42], including two empirical study-based economic evaluations [34, 40]. Seventeen publications reported on the cost-effectiveness of metabolic surgery in a population with obesity in which a subgroup of the population had T2D [43–59]. Of these 17 studies, the findings of three studies were subsequently updated or reported in more than one publication [48, 51, 57], leaving 14 studies in which a subpopulation had T2D eligible for data extraction and critical appraisal. An overview of the study selection process is presented in Fig. 1.

### Characteristics of studies

Twenty-eight of the 30 included studies were model-based economic evaluations; 14 conducted specifically in a T2D population and 14 in which a subgroup of the population had T2D. Two studies derived cost and clinical data from a single trial or observational study (i.e. empirical evidence-based studies) [34, 40].

Fifteen studies were conducted in European countries [27, 28, 36–38, 43–47, 50, 52, 55, 58, 59], four in the USA [31, 32, 35, 56], three in South America [29, 30, 49], four in Asia [39–42], three in Australia [33, 34, 53], and one in Canada [54].

Of 14 model-based economic evaluations conducted specifically in a population with T2D, eight considered a mix of metabolic surgery procedures, typically reflective of clinical practice in the reference country (Supplementary Information, Table S4) [27, 28, 30, 31, 35, 36, 38, 39]. Six studies considered RYGB or adjustable gastric banding (AGB) only [29, 32, 33, 37, 41, 42]. In general, usual care was poorly described and it is noted that country-specific clinical practice guidelines for the pharmacological management of T2D may differ. Many of the analyses were undertaken prior to the adoption of newer anti-hyperglycaemic agents such as glucagon-like peptide-1 receptor agonists (GLP-1 RA) or sodium-glucose cotransporter-2 (SGLT2) inhibitors [6].

Of 14 model-based studies in which T2D patients represented a subgroup of the population, 11 evaluations considered more than one procedure [43–47, 50, 52–55, 59]. The remaining three evaluations considered RYGB or AGB only [49, 56, 58].

An Australian within-trial cost-effectiveness analysis (cost per case of T2D remitted) with 2 years’ follow-up compared laparoscopic AGB with conventional therapy for T2D management [34]. A Chinese cost utility analysis (CUA) compared laparoscopic RYGB with usual T2D care based on data derived from a four-year observational study [40].

### Model input parameters

In general, the effect of metabolic surgery on health outcomes was modelled through measures of T2D status (changes in HbA1c, T2D remission, relapse, persistent T2D) and BMI changes (Supplementary Information, Table S4). In 13 evaluations, model predictions regarding the risk of T2D-related complications were also dependent on factors such as systolic blood pressure and/or lipid parameters [31, 32, 36–38, 43–47, 49, 55, 59].

Nine studies used long-term data from the Swedish Obesity Study either as the single source of the T2D remission rate[29, 43, 46, 47, 49, 55, 59] or to extrapolate short-term registry or trial data [33, 44]. Four studies applied a T2D remission rate to the metabolic surgery group based on evidence from randomised controlled trials (RCTs) which was extrapolated beyond the trial period [37, 39, 45, 58]. Other studies derived the T2D remission rate from literature reviews [27, 28, 30], systematic reviews or meta-analyses [31, 38, 54], cohort studies[32, 41, 42] or national datasets [35, 36, 52]. In three studies, the modelled effect of metabolic surgery on T2D was not reported [50, 53, 56].

In general, the source and/or value of all health state utilities were not clearly reported, in particular, the impact of T2D status on quality-of-life (QoL). In four studies, utility weights reflected the presence or absence of T2D alone, irrespective of BMI change [29, 33, 41, 42]. Seven studies applied utility increments per BMI unit lost [31, 32, 37, 49, 53], or assigned utility weights per BMI category [36, 52]. In thirteen studies, utility values were reported to be dependent on BMI and T2D status; however, the approach used to reflect reversion to normoglycaemia or improved glycaemic control post-surgery was not clearly described in all studies [27, 28, 30, 38, 43–47, 54, 55, 58, 59]. In one study, utility values collected from bariatric surgery patients were extrapolated beyond the data collection period [56]. Three studies did not report the utility values so it is unclear how outcomes were valued [35, 39, 50]. For the trial-based CUA, utility values were assigned based on HbA1c values (per 1% change) [40].

Skin fold removal following sustained weight loss was included in eight studies [29, 31, 43, 44, 49, 54, 56, 59]. Where reported, the modelled proportion of patients undergoing post-operative skin removal surgery varied considerably depending on the study and procedure type, from 0.8% at 2 years in two studies [43, 44], to 29% in one study [54].

### Modelling approach

All economic evaluations included in this review were cost-effectiveness analyses (CEA) or cost-utility analyses (CUA). The 14 model-based economic evaluations carried out specifically in a T2D population were CUAs [29–31, 35, 36, 38, 39, 41, 42], or presented results of CUA and CEA (cost per life year gained (LYG)) [27, 28, 32, 33, 37]. Models differed considerably in the range of diabetes-related health states considered. Eleven models assumed a binary presence or absence of T2D, or included an additional state to capture T2D improvement [27–31, 33, 35, 38, 39, 41, 42]. In three models, metabolic surgery was associated with changes in HbA1c [32, 36, 37]. Seven T2D-specific models included health states representing any diabetes-related complication, although the number of health states varied considerably, ranging from one to 10 (Supplementary Information, Table S5) [29–32, 36–38].

Among studies in which a subgroup of the population had T2D, all analyses were CUAs [43–47, 49, 50, 52–56, 58, 59], and one study presented results of CUA and CEA [49]. Health states were generally limited to macrovascular outcomes in the 14 model-based evaluations where T2D patients represented a subgroup of the overall population with obesity. In three studies, T2D-related health states were not described [50, 53, 56]. Details of the model type, perspective, time horizon and discount rate are presented in Table 1.

**Table 1:** Characteristics economic evaluations

Author (year)	Country	Type of analysis	Model type	Perspective	Time horizon	Discount rate (costs and outcomes)
**Model-based economic evaluations**						
**T2D population or sub-cohort**						
Ackroyd (2006)	Germany, UK and France	CEA; CUA	Deterministic linear algorithm	Public payer	5 years	3.5%
Anselmino (2009)	Austria, Italy and Spain	CEA; CUA	Deterministic linear algorithm	Public payer	5 years	3.5%
Assumpção (2019)	Brazil	CUA	Hybrid decision tree and markov model	Public payer	10 years	5%
Gil-Rojas (2019)	Columbia	CUA	Hybrid decision tree and 4 single-cohort markov models	Public payer	5 years	5%
Hoerger (2010)	USA	CUA	Markov (CDC-RTI Diabetes Cost-Effectiveness Model)	Not reported	Lifetime	3%
Ikramuddin (2009)	USA	CEA; CUA	Markov (CORE diabetes model)	Third party payer	Lifetime (35 years)	3%
Keating (2009b)	Australia	CEA; CUA	Markov	“Health sector”^a^	Lifetime	3%
Kim (2018)	USA	CUA	Cohort state transition model (Markov)	Private payer	5 year	3%
				Public payer (Medicare)	Lifetime	
McGlone (2020)	UK	CUA	State-transition microsimulation model	Public payer	5 years	3.5%
Pollock (2013)	UK	CEA; CUA	Markov (CORE diabetes model)	Public payer	10, 20, 30 and 40 years	3.5%
Rognoni (2020)	Italy	CUA	Markov	Public payer and societal	Lifetime	3%
Tang (2016)	China	CUA	Markov	Unclear	Not reported	5%
Viratanapanu (2019)	Thailand	CUA	Hybrid decision tree and markov model	Not reported	Lifetime	3%
Wan (2019)	China	CUA	Markov	Third party payer (insurance)	Lifetime (40 years)	5%
**Population subgroup with T2D**						
Borisenko (2018)a	England	CUA	Markov	Public payer	10 years; Lifetime	3.5%
Borisenko (2018)b	Belgium	CUA	Markov	Third-party payer	10 years; Lifetime	3% costs; 1.5% outcomes
Borisenko (2017)a	Denmark	CUA	Markov	Third-party payer	10 years; Lifetime	3%
Borisenko (2017)b	Germany	CUA	Markov	Public payer (statutory health insurance)	10 years; Lifetime	3%
Borisenko (2015)	Sweden	CUA	Markov	Public payer	Lifetime	3%
Cohen (2017)	Brazil	CEA;CUA	Markov microsimulation model	Public payer	20 years	5%
Faria (2013)	Portugal	CUA	Markov	Societal	Lifetime	3%
Gulliford (2017)	UK	CUA	Markov	Public payer	Lifetime	3%
James (2017)	Australia	CUA	Markov	Public payer	Lifetime	5%
Klarenbach (2010)	Canada	CUA	Markov	Public payer	10 years; 20 years; Lifetime	5%
McEwen (2010)	USA	CUA	Not reported	Not reported	2 years; lifetime	3%
Lucchese (2017)	Italy	CUA	Markov	Third-party payer	10 years; Lifetime	3%
Picot (2012)^b^	UK	CUA	Markov	Public payer	2, 5 and 20 years	3.5%
Sanchez-Santos (2017)	Spain	CUA	Markov	Public payer	10 years; Lifetime	3%
**Empirical-evidence based economic evaluations**						
**T2D population**						
Keating (2009a)	Australia	CEA	Trial-based economic evaluation	“Health sector”	2 years	NA
Tu (2019)	China	CUA	Parallel economic evaluation of an observational study	Unclear	4 years	Not reported

CEA, cost-effectiveness analysis; cost-utility analysis

^a^Health sector perspective comprised direct health care costs to government, private insurers, and patients

^b^Third update of a Health Technology Assessment funded by the National Institute of Health Research. Only data from the most recent update (2012) are presented

## Summary of cost-effectiveness

### Model-based studies

Results from 14 T2D-specific model-based studies (18 jurisdiction-specific analyses with 27 individual comparisons) indicated that at a WTP threshold of €20,000/QALY gained, metabolic surgery was cost-effective (14 comparisons) [27–31, 35, 37, 39, 41] or cost-saving (12 comparisons) compared with usual care (Table 2) [27, 28, 33, 36, 38, 42]. In one study, the ICER exceeded the WTP threshold of €20,000/QALY, but would still be considered cost-effective at a WTP threshold of €45,000/QALY gained [32].

**Table 2:** Adjusted incremental cost-effectiveness ratios

Author (year)	Country	Cost year	Unadjusted ICER	Adjusted ICER (€/QALY or €/case of T2D remitted)^a,b^
**Model-based economic evaluations**				
**T2D population or sub-cohort**				
Bariatric surgery versus usual care^c^				
Gil-Rojas (2019)	Columbia	2016	6,194,899 COP	4,531/QALY
McGlone (2020)	UK	2018	Dominant	Dominant
Rognoni (2020)	Italy	2018	Dominant	Dominant
Gastric banding versus usual care				
Ackroyd (2006)	Germany	2005	Dominant	Dominant
	France	2005	Dominant	Dominant
	UK	2005	£1,929	3,269/QALY
Anselmino (2009)	Austria	2009	Dominant	Dominant
	Italy	2009	Dominant	Dominant
	Spain	2009	€1,456	2,104/QALY
Hoerger (2010)	USA	2005	USD $ 12,098^d^	15,848/QALY
Keating (2009)b	Australia	2006	Dominant	Dominant
Kim (2018)	USA	2014	US $7789^d^	7,577/QALY
Pollock (2013)	UK	2010	£3,602	5,275/QALY
Gastric bypass versus usual care				
Ackroyd (2006)	Germany	2005	Dominant	Dominant
	France	2005	Dominant	Dominant
	UK	2005	£1,517	2,571/QALY
Anselmino (2009)	Austria	2009	Dominant	Dominant
	Italy	2009	Dominant	Dominant
	Spain	2009	€2,664	3,850/QALY
Assumpção (2019)	Brazil	2015	Int $1,820	1,278/QALY
Hoerger (2010)	USA	2005	USD $9,172^d^	12,015/QALY
Ikramuddin (2009)	USA	2007	US $21,973	26,502/QALY
Kim (2018)	USA	2014	$7,844^d,e^	7,630/QALY
Tang (2016)	China	Not reported^f^	Int $451^g^	116/QALY
Viratanapanu (2019)	Thailand	2017	26,908 THB	1,863/QALY
Wan (2019)	China	2015	Dominant	Dominant
Sleeve gastrectomy versus usual care				
Tang (2016)	China	Not reported^f^	Int $361^g^	92/QALY
**T2D subpopulation**				
Bariatric surgery versus usual care^‡^				
Borisenko (2018)a	England	2015	Dominant	Dominant
Borisenko (2018)b	Belgium	2012	Dominant	Dominant
Borisenko (2017)a	Denmark	2012	Dominant	Dominant
Borisenko (2017)b	Germany	2012	Dominant	Dominant
Borisenko (2015)	Sweden	2012	Dominant	Dominant
Gulliford (2017)	UK	2013	£6,176	8,296/QALY
Lucchese(2017)	Italy	2013	Dominant	Dominant
Sanchez-Santos (2017)	Spain	2017	Dominant	Dominant
Gastric banding versus usual care				
Faria (2013)	Portugal	Not reported^f^	€1,810^d^	2,845/QALY
James (2017)	Australia	2015	Dominant	Dominant
Picot (2012)	UK	2009	US $8831	2,462/QALY
Gastric bypass versus usual care				
Cohen (2017)	Brazil	Not reported^f^	Dominant	Dominant
Faria (2013)	Portugal	Not reported^f^	Dominant	Dominant
James (2017)	Australia	2015	Dominant	Dominant
Klarenbach (2010)	Australia	2009	Dominant	Dominant
McEwen (2010)	USA	2007	USD $8,831	10,651/QALY
Sleeve gastrectomy versus usual care				
James (2017)	Australia	2015	Dominant	Dominant
**Empirical evidence-based economic evaluations**				
**T2D population**				
Gastric banding versus usual care				
Keating (2009)^a^	Australia	2006	16,600	16,554/case of T2D remitted
Gastric bypass versus usual care				
Tu (2019)	China	2013	¥ 125,836	32,270/QALY

COP, Colombian pesos; ICER, incremental cost-effectiveness ratio; QALY, quality-adjusted life year; THB,hai baht; USA, United States of America

^a^Where multiple time horizons were used, results for the longest time horizon are presented

^b^Adjusted ICER is defined as inflation of a context-specific ICER using country-specific consumer price indices (CPI) to a common cost year (2020), prior to conversion to a common currency (Irish Euro) using purchasing power parities (PPPs). PPPs are indicators of price level differences between countries. Even in countries using a common currency (e.g. Euro), differences in local economies influence the price of products

^c^Bariatric surgery comprises a mix of surgeries, typically based on the mix of surgeries in use in clinical practice in the index country

^d^Where ICERs were presented by subgroup only, a simple or weighted average ICER was calculated

^e^A weighted-average ICER for laparoscopic Roux-en-Y gastric bypass is presented. ICERs for open RYGB are presented in the Supplementary Information, Table S7

^f^Where the cost year was not reported, the average interval between the cost and publication year in other included studies (3 years) was assumed

^g^ICERs were not presented in the original study. ICERs were calculated based on the incremental costs and QALYs provided

**Fig. 2:**
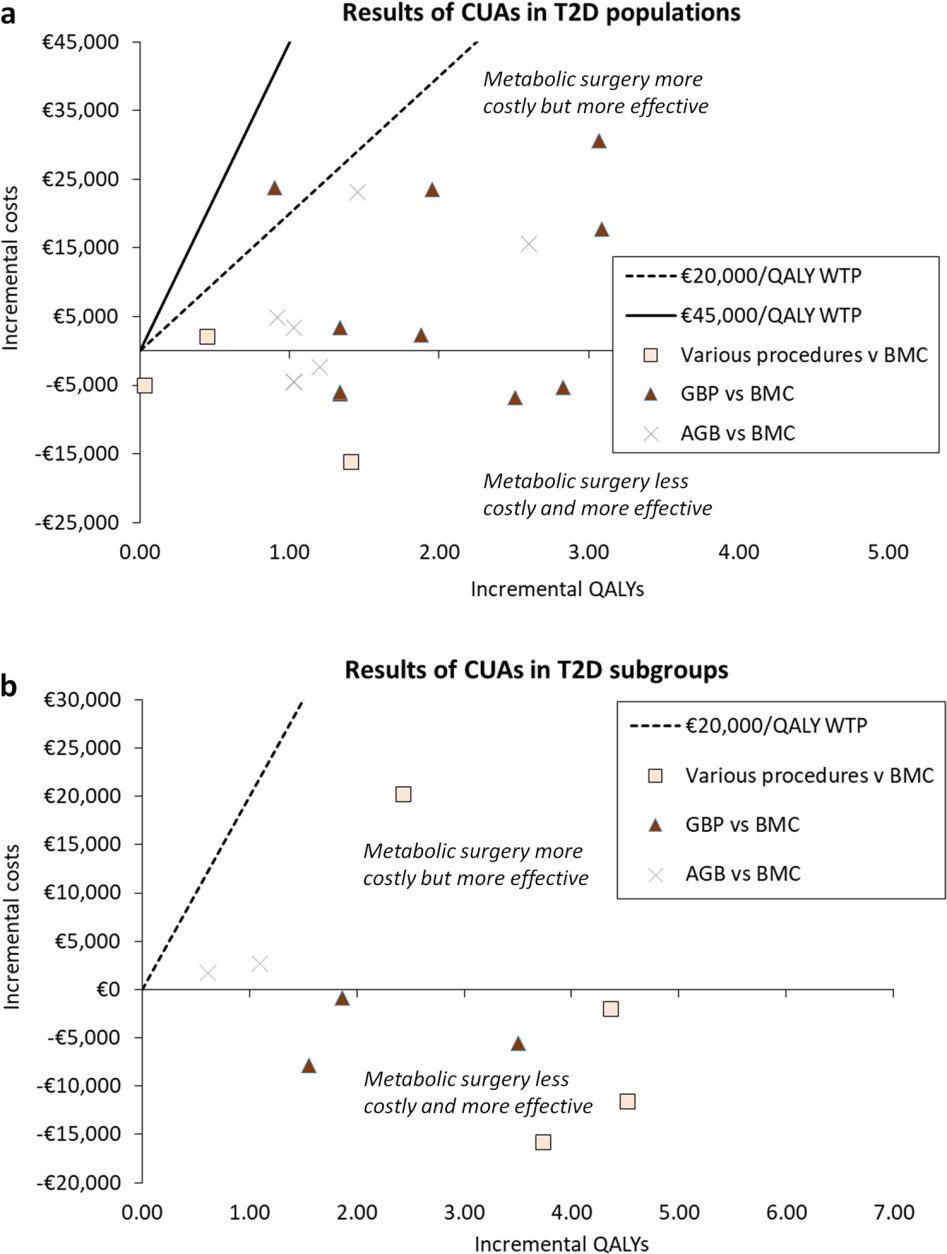
Cost-effectiveness plane^a,b,c^. Panel a shows ICERs for CUAs in a T2D population. Panel b shows ICERs for CUAs in a T2D subpopulation. Results can be interpreted with reference to WTP thresholds of €20,000/QALY gained or €45,000/QALY gained. Key: AGB, adjustable gastric band; BMC, best medical care; CUA, cost utility analysis; GBP, gastric bypass; WTP, willingness-to-pay; QALY, quality-adjusted life year. ^a^For some studies, the incremental costs and incremental QALYs used to calculate the ICER were not reported [28, 45–47, 53, 55, 56], therefore, ICERs could not be plotted on the cost-effectiveness plane. ^b^ICERs for empirical-evidence based economic evaluations are not shown. ^c^The uncertainty surrounding ICERs is not shown (data not reported)

Among 14 studies in which a subgroup of the population had T2D (14 jurisdiction-specific analyses with 17 individual comparisons), metabolic surgery was the dominant strategy (less costly and more effective) in 13 comparisons (Table 2) [43–47, 49, 50, 53–55, 59]. Surgery was cost-effective in four comparisons at a WTP threshold of €20,000/QALY, with adjusted ICERs ranging from €2,462 to €10,651 per QALY gained [50, 52, 56, 58]. In one of these studies the outcome varied depending on the BMI category, however, on average, gastric bypass and gastric banding were cost-saving or cost-effective, respectively [50]. In general, results were sensitive to the modelled time horizon; better outcomes were observed over longer time horizons. Figure 2 shows incremental costs (in 2020 Irish Euro) and QALYs of model-based CUAs on a cost-effectiveness plane. For ICERs in the northeast quadrant (more costly, more effective), all ICERs fall below the WTP threshold of €45,000 per QALY gained.

Across all model-based economic evaluations (*n* = 28), 13 studies estimated the cost-effectiveness of surgery according to population or healthcare system characteristics including BMI category (*n* = 10) [35, 38, 43–47, 50, 55, 59], sex (*n* = 8) [35, 43–47, 55, 59], age (*n* = 2) [31, 53], duration of T2D (*n* = 1) [31], and length of time on the waiting list (*n* = 2) [43, 49]. Eight studies reported that metabolic surgery was the dominant strategy irrespective of the BMI category over a life-time time horizon [38, 43–47, 55, 59]. In one study, the ICER decreased with increasing BMI,[35], and in another, the lowest ICER was observed in those with an intermediate BMI (40–50 kg/m^2^) [50]. In line with recent changes to metabolic surgery eligibility criteria to expand access to those with lower levels of obesity and obesity-related comorbidities [60, 61], 13 model-based studies included populations with a BMI of 30–34.9 kg/m^2^.[33, 35, 37, 38, 43–47, 50, 55, 58, 59] Metabolic surgery was reported to be cost-effective or cost-saving in patients with class I obesity (BMI 30–34.9 kg/m^2^) and T2D in all of these studies. In one study, ICERs for both gastric bypass and gastric banding were more favourable in younger patients [31]. The same study conducted subgroup analysis according to duration of T2D, finding that metabolic surgery was most cost-effective in younger patients with newly diagnosed T2D (< 5 years after diagnosis) and least cost-effective in older patients with established T2D (≥ 10 years after diagnosis), which was attributed to the higher T2D remission rate in those with a shorter duration of disease [31]. Sex had no to little effect on ICERs. Two studies examined the impact of length of time on the waiting list prior to surgery on cost-effectiveness. In both analyses, delays in accessing surgery were associated with increasing costs and decreasing benefits, highlighting the importance of early access to surgery [43, 49].

### Empirical evidence-based studies

Of the 30 included studies, two were based on a single RCT or observational study. In a two-year trial-based CEA, laparoscopic AGB was reported to be cost-effective compared with usual care in a population with recent-onset T2D (Table 2) [34]. In a four-year observational study based CUA in which RYGB was compared with usual care, surgery was not cost-effective at the €20,000/QALY threshold, but would be considered cost-effective at a threshold of €45,000/QALY [40]. Of note, the time horizon of this analysis was likely too short for the initial costs of surgery to have been offset by the long-term benefits.

### Sensitivity analysis in included studies

For studies carried out specifically in a T2D population, where one-way sensitivity analysis was undertaken, the results were largely robust to variations of the tested input parameters. However, uncertainty associated with the following parameters led to the most substantial change in the estimated ICERs: utility weights [29, 31, 32, 36, 42], the impact of surgery or treatment on HbA1c values or T2D remission [36, 41], and a number of cost parameters including the cost of usual care [29, 31, 36, 39, 41, 42], surgery [29, 31, 39, 41, 42], diabetes-related complications (stroke) [30] and follow-up care [31]. For studies in which T2D patients were a subgroup of an overall population with obesity, one-way sensitivity analysis was generally undertaken in the context of the overall population, therefore the applicability of the results to the sub-population with T2D is unclear. In some of these evaluations, a diagnosis of T2D or T2D treatment costs were among the most influential parameters during one-way sensitivity analysis [44, 45, 47, 55]. Overall, 21 studies investigated methodological or structural uncertainty through scenario analysis [27, 28, 32, 33, 35–38, 43–47, 49, 52–56, 58, 59], eight specifically in a T2D population [27, 28, 32, 33, 35–38]. In general, results remained robust after changes to the model structure or inputs. In T2D-specific models, only three scenarios yielded an ICER that would exceed the WTP threshold adopted in the original study, namely a “worst-case scenario”, excluding the negative impact of increased BMI on quality of life and decreasing the time horizon to five or 10 years [32, 37].

### Methodological quality

**Fig. 3:**
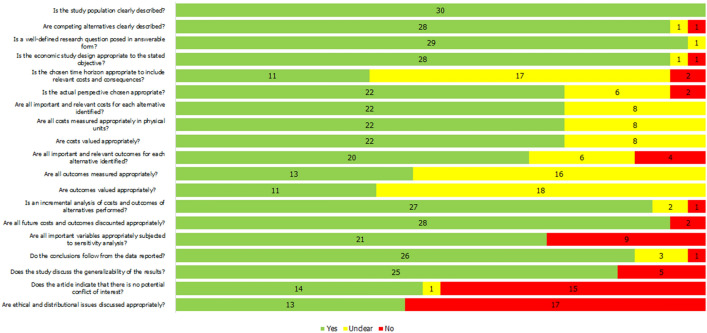
Methodological quality assessment of economic evaluations using CHEC-list

The methodological quality of included economic evaluations (*n* = 30) was variable. Studies were categorised as high (*n* = 15) [31, 32, 36, 37, 43–47, 49, 52, 54, 55, 58, 59], moderate (*n* = 5)[27, 30, 35, 38, 53] or low (*n* = 10)[28, 29, 33, 34, 39–42, 50, 56] quality. The most common issues related to insufficient reporting of input parameters or the modelling approach. Of the 30 studies included, only six studies modelled both microvascular and macrovascular health states which may have implications for the face validity of modelled outcomes [31, 32, 36–38, 50]. Inclusion of a limited number of diabetes-related diseases may bias the analysis against the intervention owing to a failure to account for cost savings of diabetes-related complications avoided. In addition, several studies used outcome data from surgical procedures that no longer reflect clinical practice [29, 33, 34, 37, 43, 44, 46, 47, 49, 54, 55, 58, 59]. With consideration to the chronic nature of T2D, it is unlikely that shorter time horizons (up to five years) fully capture the impact of surgery on diabetes-related morbidity and mortality, nor the potential for relapse or long-term post-surgical complications [27, 28, 30, 34, 36, 40]. Given uncertainty regarding the long-term effects of surgery due to the limited amount of high-quality evidence with long-term, clinically relevant follow-up, estimation of cost-effectiveness over two or more time horizons, adopted in eleven models, was considered to be the most appropriate approach [35, 37, 44–47, 54–56, 58, 59]. Assessment of methodological, structural or parameter uncertainty was considered inadequate in nine studies (Fig. 3) [27, 28, 34, 35, 39–41, 50, 56].

### Transferability

Evidence of clinical and thus cost-effectiveness may not be transferable from one context to another given differences in population and healthcare system characteristics including differences in obesity-related risk among ethnic groups, healthcare system structure and financing, and the need for quality assurance processes to ensure optimal outcomes [62]. In addition to context-specific considerations, heterogeneity in parameter values and structural shortcomings such as the time horizon and health states modelled limit the transferability to other contexts. Although no studies were considered directly applicable to other settings, 17 studies were considered partially applicable (Supplementary Information, Fig. S1) [30–32, 36–38, 43–47, 49, 52, 54, 55, 58, 59].

While bariatric or metabolic surgery have traditionally been limited to high income countries, emerging evidence of cost-effectiveness in middle income countries may support expansion of its use in these contexts [29, 30, 39–42, 49], many of which are experiencing a dramatic increase in obesity prevalence and are thus in need of effective interventions to manage obesity and its complications [63].

For the purposes of this systematic review, ICERs were adjusted (see Methods) to facilitate comparison across studies and interpretation of the evidence. There is no universally accepted, gold standard method to identify appropriate WTP thresholds. Variation in methods and economic conditions result in differences in the WTP thresholds adopted across countries. Nevertheless, the thresholds adopted in this review are broadly consistent with those used elsewhere in Europe [25, 26]. Therefore, the results are likely applicable to other European contexts. ICERs and associated WTP thresholds as reported by the primary economic evaluations are presented in Supplementary Information 3, Table S6. Importantly, interpretation of the findings with reference to the current Irish context did not change the conclusions of the original economic evaluations.

## Discussion

The cost-effectiveness of metabolic surgery for the treatment of comorbid T2D and obesity was systematically reviewed, including evidence from 30 cost-effectiveness analyses performed across multiple jurisdictions. Metabolic surgery was reported to be cost-saving or cost-effective in the base case analyses at a WTP threshold of €20,000/QALY gained in 28 studies. In two studies, the ICER exceeded a WTP threshold of €20,000/QALY, but would still be considered cost-effective at a WTP threshold of €45,000/QALY [32, 40].

While three previous systematic reviews have evaluated the cost-effectiveness of bariatric surgery for the treatment of obesity, these reviews did not focus specifically on the costs and benefits of metabolic surgery for the treatment of T2D, which differ substantially from the population with obesity both in terms of the cost of usual care and the clinical benefits of surgery [15–17]. The results of the current systematic review are in agreement with those of previous systematic reviews; bariatric surgery is a cost-effective approach to treating obesity, particularly in populations with comorbid T2D. Since publication of the previous systematic reviews, at least nine cost-effectiveness models have been published [29, 30, 35, 36, 38, 40–42, 44], eight specifically in T2D populations [29, 30, 35, 36, 38, 40–42], consistent with the shift in the clinical focus of bariatric surgery towards increased consideration of the potential for surgery to treat obesity-related comorbidities, such as T2D, as opposed to weight-loss alone.

Based on the range of surgical procedures included in the identified economic evaluations (LAGB, SG, RYGB and biliopancreatic diversion (BPD)) metabolic surgery is cost-effective or cost-saving irrespective of the procedure used. At present, RYGB and SG are the most commonly performed bariatric surgeries globally [14]. Evidence from RCTs indicates that remission rates are comparable between RYGB and SG [64–66]. Thus, a change in the proportion of RYGB or SG in use in favour of the other is unlikely to influence the cost-effectiveness of metabolic surgery. The use of laparoscopic one anastomosis gastric bypass (LOAGB) is increasing, but still accounts for a small proportion of all surgeries performed [14, 67]. Evaluation of the cost-effectiveness of newer procedures such as LOAGB will be dependent on evidence of clinical effectiveness and safety.

In order to maximise the health and economic benefits of surgery, delays in accessing surgery should be minimised, as demonstrated by the increased cost-effectiveness of surgery in those with recent-onset T2D and the negative impact of delays in surgery provision on outcomes of surgery [31, 43, 49]. As the prevalence of obesity and obesity-related complications increases worldwide, demand for bariatric and metabolic surgery is likely to continue to exceed supply [68–70]. The ability to identify subgroups of the population more likely to benefit from metabolic surgery is important to inform optimal selection of surgical candidates and allocation of scarce resources. In general, the results of subgroup analyses indicated that surgery was likely to be cost-effective irrespective of BMI category or sex. However, it is increasingly recognised that BMI is a poor predictor of benefit from surgery in patients with comorbid T2D and obesity [71]. In recent years, professional organisations have recommended a change from BMI-based eligibility criteria to a comorbidity-based approach to candidate selection in recognition of the considerable inter-individual variability in the adverse health consequences of obesity as defined by BMI [13, 60, 61, 72]. The results of several modelling studies indicated that metabolic surgery may be cost-effective even for those with class I obesity and T2D, to whom it has not traditionally been offered [33, 35, 37, 38, 43–47, 50, 55, 58, 59]. Only one study presented ICERs according to duration of T2D, reporting the best value for money in younger patients with shorter duration of disease [31]. Further investigation of diabetes-specific characteristics, such as the duration or severity of T2D, may provide a better indicator of T2D subgroups for whom metabolic surgery may yield the greatest clinical and economic benefits.

The accuracy of model predictions is influenced by the choices around key structural aspects and input parameters [73, 74]. As high-quality evidence from long-term studies is limited, the majority of economic evaluations in this systematic review extrapolated clinical data from medium-term RCTs or long-term observational evidence from the Swedish Obese Subjects (SOS) study [75–77]. The surgical methods used in these studies (e.g. vertical banded gastroplasty, LAGB or use of the open surgical approach) may not reflect current surgical practice and may therefore produce inaccurate results. However, this may be considered a conservative approach given that LAGB procedures have been associated with lower T2D remission and higher complications rates in comparison with current clinical practice [61, 78]. Modelling over longer time horizons requires increasing dependence on assumptions due to limitations in the evidence base, however, the shorter time horizons adopted in some studies may produce biased outcomes by failing to capture the costs and effects of long-term surgical or diabetes-related complications. RCTs of metabolic surgery have, to date, been underpowered to detect differences in the risk of macrovascular events, a major source of T2D-related healthcare expenditure [79, 80]. Moreover, important feasibility issues including the need for targeted pre-operative screening, challenging retention in the usual care arm, and funding mean that large multicentre trials powered to detect differences in the risk of cardiovascular events are unlikely to be possible [81]. Information regarding the long-term effects of metabolic surgery on the progression or development of cardiovascular complications may need to come from carefully controlled observational studies.

The impact of metabolic surgery on health-related QoL is multifactorial which presents challenges for the estimation of health gains [82, 83]. The disutility associated with excess adiposity or diabetes-related complications is well documented [82, 84], but the potential impact of T2D remission on utility is unclear. There was considerable variation in approaches to estimating the utility gain associated with improvements in glycaemic control. In studies where utility gains were based only on changes in BMI, it is likely that the potential benefit of metabolic surgery on QoL has been underestimated. The relationship between T2D and QoL is complex; changes in glycaemic control, insulin use and body weight are all likely to affect QoL in patients with T2D and obesity [85]. Although much of the benefit of improvements in glycaemic control is in the prevention of long-term diabetes-related complications, glycaemic control is likely to affect some aspects of daily living, for example, the need for daily planning of injection times, dose adjustment or monitoring of glycaemic control (where indicated) for insulin-treated T2D [85]. Further research is necessary to determine how changes in glycaemic control impact QoL in patients with comorbid T2D and obesity.

Only eight models incorporated the cost of excess skin removal following sustained weight-loss, which may be related to availability within the public healthcare system in the reference country. In clinical practice, it is likely that an imbalance exists between the number of people who desire excess skin removal surgery and those who actually receive it [86]. Inclusion of additional surgeries to remove excess skin in the surgery group, where indicated, would result in additional costs, but may yield clinical benefits and improvements in QoL [87].

The rising prevalence of obesity and obesity-related diseases, in particular T2D, is among the greatest challenges facing health systems [70]. Worldwide, there is an unmet need for bariatric and metabolic surgery, which is likely to continue based on current epidemiological trends [70, 72]. Clinicians and policy makers should ensure that metabolic surgery is appropriately considered in the management of patients with comorbid T2D and obesity, without unintentionally redirecting resources away from existing bariatric surgery services which could contribute to exacerbation of health inequities. Future economic evaluations should the explore the budgetary implications, resources and operational considerations of introducing a metabolic surgery service in addition to a bariatric surgery service to ensure that these services work synergistically to ensure adequate capacity to meet increasing demand.

## Strengths and limitations

This systematic review has several strengths. Given that metabolic surgery has only been recommended for the treatment of T2D in recent years [13], broad eligibility criteria were applied in order to capture studies in which only a subgroup of the population had T2D. Thus, this systematic review provides a comprehensive overview of all published economic evaluations considering the cost-effectiveness of metabolic surgery in patients with T2D. Secondly, the quality and transferability of the evidence has been assessed to determine the validity and relevance of modelled outcomes.

Despite these strengths, there are a number of limitations associated with this systematic review. Exclusion of non-English language studies represents a source of bias; however, given the range of countries and contexts covered and the consistency of the findings, this bias is unlikely to have impacted the interpretation of the evidence. Secondly, the validity and relevance of modelled outcomes and cost data are presented in the Irish context. However, while the factors limiting transferability may vary in other settings, methodological shortcomings including the number of T2D-related health states modelled and uncertainty regarding the utility gain associated with T2D remission represent universally relevant limitations. It is acknowledged that restricting the perspective to specific local circumstances may be perceived as a barrier to generalisation of results to other contexts. However, of note, interpretation of the findings of this review with reference to the current Irish context did not change the conclusions of the primary economic evaluations because studies generally found that metabolic surgery was cost-saving or highly cost-effective compared with best medical care (Supplementary Information, Table S6). Thirdly, for studies where subgroup-specific ICERs were presented, a simple or weighted average ICER, was calculated. While ICERs for individual subgroups typically varied marginally [31, 35, 38, 43, 44], in one study the findings varied considerably between subgroups, thus, the overall ICER should be interpreted with caution [50]. While estimation of subgroup-specific ICERs may highlight subgroups with greater capacity to benefit from surgery, it was unclear if differential subgroup effects were attributable to true heterogeneity in treatment benefits and costs between subgroups or were as a result of modelled assumptions. Finally, since the literature search was undertaken, the evidence base has continued to expand, with the publication of one additional study meeting the inclusion criteria. Consistent with the included studies, metabolic surgery was reported to be cost-saving over a lifetime time horizon in this analysis undertaken in the UK [88]. Given the strength of the evidence base, the broad range of contexts considered and the consistency of the findings, a full update of this systematic review prior to publication was not considered necessary, as it was considered unlikely that an update would change the conclusions of this systematic review at this time. However, an important limitation of the underlying clinical effectiveness data is that many RCTs and observational studies were undertaken prior to the widespread use of a number of new, effective anti-hyperglycaemic medication classes with additional benefits in terms of weight loss and cardiovascular risk factor control when compared with established anti-hyperglycaemic agents [6].

## Conclusions

Despite the limitations of the included economic evaluations, metabolic surgery may be considered a cost-effective intervention for patients with comorbid T2D and obesity, or cost-saving if outcomes are modelled over longer time horizons. Addressing identified research gaps, including the scarcity of long-term, high-quality evidence to determine the impact of metabolic surgery on diabetes-related complications and uncertainty regarding the impact of metabolic surgery on quality of life, will allow more accurate prediction of the lifetime costs and consequences associated with metabolic surgery. As the prevalence of obesity and obesity-related diseases increases worldwide, significant investment will be required to ensure that service provision is adequate to meet demand.

## Supplementary Information

Below is the link to the electronic supplementary material.

Supplementary file1 (DOCX 156 kb)

## Data Availability

Data sharing is not applicable to this article as new data were not created.

## References

[1] Khan MAB, Hashim MJ, King JK, Govender RD, Mustafa H, Al Kaabi J (2020). Epidemiology of type 2 diabetes—global burden of disease and forecasted trends, (in eng). J. Epidemiol. Glob. Health.

[2] Lin X (2020). Global, regional, and national burden and trend of diabetes in 195 countries and territories: an analysis from 1990 to 2025. Sci. Rep..

[3] Seuring T, Archangelidi O, Suhrcke M (2015). The economic costs of type 2 diabetes: a global systematic review, (in eng). Pharmacoeconomics.

[4] Artime E, Romera I, Díaz-Cerezo S, Delgado E (2021). Epidemiology and economic burden of cardiovascular disease in patients with type 2 diabetes mellitus in Spain: a systematic review, (in eng). Diabetes Ther..

[5] Bommer C (2018). Global economic burden of diabetes in adults: projections from 2015 to 2030, (in eng). Diabetes Care.

[6] American Diabetes Association Professional Practice Committee: Pharmacologic approaches to glycemic treatment: standards of medical care in diabetes—2022. Diabetes Care **45**( Supplement_1), S125–S143 (2022) 10.2337/dc22-S009 34964831

[7] American Diabetes Association Professional Practice Committee: Facilitating behavior change and well-being to improve health outcomes: standards of medical care in diabetes—2022. Diabetes Care **45**(Supplement_1), S60–S82 (2022) 10.2337/dc22-S005 34964866

[8] American Diabetes Association Professional Practice Committee: Obesity and weight management for the prevention and treatment of type 2 diabetes: standards of medical care in diabetes—2022. Diabetes Care **45**(Supplement_1), S113–S124 (2022) 10.2337/dc22-S008 34964843

[9] American Diabetes Association Professional Practice Committee: Cardiovascular disease and risk management: standards of medical care in diabetes—2022. Diabetes Care **45**(Supplement_1), S144–S174 (2022) 10.2337/dc22-S010 34964815

[10] Fried M (2014). Interdisciplinary European guidelines on metabolic and bariatric surgery, (in eng). Obes Surg.

[11] Rubin JK, Hinrichs-Krapels S, Hesketh R, Martin A, Herman WH, Rubino F (2016). Identifying barriers to appropriate use of metabolic/bariatric surgery for type 2 diabetes treatment: policy lab results, (in eng). Diabetes Care.

[12] Luck-Sikorski C, Jung F, Dietrich A, Stroh C, Riedel-Heller SG (2019). Perceived barriers in the decision for bariatric and metabolic surgery: results from a representative study in Germany. Obes. Surg..

[13] Rubino F (2016). Metabolic surgery in the treatment algorithm for type 2 diabetes: a joint statement by international diabetes organizations, (in eng). Diabetes Care.

[14] Ramos, A., et al.: 5th IF20 Global Registry Report, International Federation for the Surgery of Obesity and Metabolic Diseases (IFSO) (2019)

[15] Xia Q, Campbell JA, Ahmad H, Si L, de Graaff B, Palmer AJ (2020). Bariatric surgery is a cost-saving treatment for obesity-A comprehensive meta-analysis and updated systematic review of health economic evaluations of bariatric surgery, (in eng). Obes. Rev..

[16] Louwagie, P., et al.: Bariatric surgery: an HTA report on the efficacy, safety and cost-effectiveness, Belgian Health Care Knowledge Centre (KCE) KCE Reports 316. D/2019/10.273/44, Brussels (2019)

[17] Campbell JA, Venn A, Neil A, Hensher M, Sharman M, Palmer AJ (2016). Diverse approaches to the health economic evaluation of bariatric surgery: a comprehensive systematic review, (in eng). Obes. Rev..

[18] Rubino F, Shukla A, Pomp A, Moreira M, Ahn SM, Dakin G (2014). Bariatric, metabolic, and diabetes surgery: what's in a name?, (in eng). Ann. Surg..

[19] Moher D, Liberati A, Tetzlaff J, Altman DG (2009). Preferred reporting items for systematic reviews and meta-analyses: the PRISMA statement. BMJ.

[20] Mandrik O (2021). Critical appraisal of systematic reviews with costs and cost-effectiveness outcomes: an ISPOR good practices task force report. Value Health.

[21] Scottish Intercollegiate Guidelines Network: Search filters. https://www.sign.ac.uk/what-we-do/methodology/search-filters/ (2020)

[22] Evers S, Goossens M, de Vet H, van Tulder M, Ament A (2005). Criteria list for assessment of methodological quality of economic evaluations: consensus on Health Economic Criteria, (in eng). Int. J. Technol. Assess. Health Care.

[23] Jaime-Caro J (2014). Questionnaire to assess relevance and credibility of modeling studies for informing health care decision making: an ISPOR-AMCP-NPC Good Practice Task Force report, (in eng). Value Health.

[24] Health Information and Quality Authority: Guidelines for the Economic Evaluation of Health Technologies in Ireland. https://www.hiqa.ie/reports-and-publications/health-technology-assessment/guidelines-economic-evaluation-health (2020)

[25] Woods B, Revill P, Sculpher M, Claxton K (2016). Country-level cost-effectiveness thresholds: initial estimates and the need for further research, (in eng). Value Health.

[26] Iino H, Hashiguchi M, Hori S (2022). Estimating the range of incremental cost-effectiveness thresholds for healthcare based on willingness to pay and GDP per capita: a systematic review. PLoS ONE.

[27] Ackroyd R, Mouiel J, Chevallier JM, Daoud F (2006). Cost-effectiveness and budget impact of obesity surgery in patients with type-2 diabetes in three European countries, (in English). Obes. Surg..

[28] Anselmino M, Bammer T, Fernandez-Cebrian JM, Daoud F, Romagnoli G, Torres A (2009). Cost-effectiveness and budget impact of obesity surgery in patients with type 2 diabetes in three European countries(II). Obes. Surg..

[29] Assumpção RP (2019). Cost-utility of gastric bypass surgery compared to clinical treatment for severely obese with and without diabetes in the perspective of the Brazilian public health system, (in English). Obes. Surg..

[30] Gil-Rojas Y, Garzón A, Lasalvia P, Hernández F, Castañeda-Cardona C, Rosselli D (2019). Cost-effectiveness of bariatric surgery compared with nonsurgical treatment in people with obesity and comorbidity in Colombia, (in English). Value Health Region. Issues.

[31] Hoerger TJ, Zhang P, Segel JE, Kahn HS, Barker LE, Couper S (2010). Cost-effectiveness of bariatric surgery for severely obese adults with diabetes, (in English). Diabetes Care.

[32] Ikramuddin S, Klingman D, Swan T, Minshall ME (2009). Cost-effectiveness of Roux-en-Y gastric bypass in type 2 diabetes patients. Am. J. Manag. Care.

[33] Keating CL (2009). Cost-effectiveness of surgically induced weight loss for the management of type 2 diabetes: modeled lifetime analysis, (in English). Diabetes Care.

[34] Keating CL, Peeters A, Dixon JB, Playfair J, Moodie ML, O'Brien PE (2009). Cost-efficacy of surgically induced weight loss for the management of type 2 diabetes, (in English). Diabetes Care.

[35] Kim DD, Arterburn DE, Sullivan SD, Basu A (2018). Economic value of greater access to bariatric procedures for patients with severe obesity and diabetes, (in English). Med. Care.

[36] McGlone ER (2020). Bariatric surgery for patients with type 2 diabetes mellitus requiring insulin: clinical outcome and cost-effectiveness analyses. PLoS Med..

[37] Pollock RF, Muduma G, Valentine WJ (2013). Evaluating the cost-effectiveness of laparoscopic adjustable gastric banding versus standard medical management in obese patients with type 2 diabetes in the UK. Diabetes Obes. Metab..

[38] Rognoni C, Armeni P, Tarricone R, Donin G (2020). Cost–benefit analysis in health care: the case of bariatric surgery compared with diet, (in English). Clin. Therapeut..

[39] Tang Q (2016). Cost-effectiveness of bariatric surgery for type 2 diabetes mellitus, (in English). Med. (US).

[40] Tu Y (2019). Cost-utility of laparoscopic Roux-en-Y gastric bypass in Chinese patients with type 2 diabetes and obesity with a BMI ≥ 27.5 kg/m^2^: a multi-center study with a 4-year follow-up of surgical cohort, (in English). Obes. Surg..

[41] Viratanapanu I (2019). Cost-effectiveness evaluation of bariatric surgery for morbidly obese with diabetes patients in Thailand, (in English). J. Obes..

[42] Wan B (2019). Cost-effectiveness of bariatric surgery versus medication therapy for obese patients with type 2 diabetes in china: a Markov analysis, (in English). J. Diabetes Res..

[43] Borisenko O (2015). Bariatric surgery can lead to net cost savings to health care systems: results from a comprehensive European decision analytic model. Obes. Surg..

[44] Borisenko O, Lukyanov V, Ahmed AR (2018). Cost-utility analysis of bariatric surgery, (in English). Br. J. Surg..

[45] Borisenko O, Lukyanov V, Debergh I, Dillemans B (2018). Cost-effectiveness analysis of bariatric surgery for morbid obesity in Belgium, (in English). J. Med. Econ..

[46] Borisenko O, Lukyanov V, Johnsen SP, Funch-Jensen P (2017). Cost analysis of bariatric surgery in Denmark made with a decision-analytic model. Danish Med. J..

[47] Borisenko O, Mann O, Dupree A (2017). Cost-utility analysis of bariatric surgery compared with conventional medical management in Germany: a decision analytic modeling. BMC Surg..

[48] Clegg A, Colquitt J, Sidhu M, Royle P, Walker A (2003). Clinical and cost effectiveness of surgery for morbid obesity: a systematic review and economic evaluation, (in English). Int. J. Obes. Rev..

[49] Cohen RV, Luque A, Junqueira S, Ribeiro RA, Le Roux CW (2017). What is the impact on the healthcare system if access to bariatric surgery is delayed?, (in English). Surg. Obes. Relat. Dis..

[50] Faria GR, Preto JR, Costa-Maia J (2013). Gastric bypass is a cost-saving procedure: results from a comprehensive markov model, (in English). Obes. Surg..

[51] Gulliford, M.C., et al.: Costs and outcomes of increasing access to bariatric surgery for obesity: cohort study and cost-effectiveness analysis using electronic health records, NIHR Journals Library 2016. https://www.journalslibrary.nihr.ac.uk/hsdr/hsdr04170/#/abstract (2016) 27253004

[52] Gulliford MC (2017). Costs and outcomes of increasing access to bariatric surgery: cohort study and cost-effectiveness analysis using electronic health records. Value Health.

[53] James R, Salton RI, Byrnes JM, Scuffham PA (2017). Cost-utility analysis for bariatric surgery compared with usual care for the treatment of obesity in Australia. Surg. Obes. Relat. Dis..

[54] Klarenbach, S., et al.: Bariatric surgery for severe obesity: systematic review and economic evaluation. https://www.cadth.ca/media/pdf/H0485_Bariatric_Surgery_for_Severe_Obesity_tr_e.pdf (2010). Accessed 3 Mar 2021

[55] Lucchese M (2017). Cost-utility analysis of bariatric surgery in Italy: results of decision-analytic modelling. Obes. Facts.

[56] McEwen LN, Coelho RB, Baumann LM, Bilik D, Nota-Kirby B, Herman WH (2010). The cost, quality of life impact, and cost-utility of bariatric surgery in a managed care population, (in eng). Obes. Surg..

[57] Picot J (2009). The clinical effectiveness and cost-effectiveness of bariatric (weight loss) surgery for obesity: a systematic review and economic evaluation. Health Technol. Assess. (Winchester, Engl.).

[58] Picot J, Jones J, Colquitt JL, Loveman E, Clegg AJ (2012). Weight loss surgery for mild to moderate obesity: a systematic review and economic evaluation. Obes. Surg..

[59] Sanchez-Santos R (2018). Bariatric surgery versus conservative management for morbidly obese patients in Spain: a cost-effectiveness analysis. Expert Rev. Pharmacoecon. Outcomes Res..

[60] Obesity Management for the Treatment of Type 2 Diabetes: Standards of Medical Care in Diabetes-2021, (in eng). Diabetes Care **44**(Suppl 1), S100–s110 (2021) 10.2337/dc21-S008 33298419

[61] Di Lorenzo N (2020). Clinical practice guidelines of the European Association for Endoscopic Surgery (EAES) on bariatric surgery: update 2020 endorsed by IFSO-EC, EASO and ESPCOP. Surg. Endosc..

[62] Metabolic and Bariatric Surgery Accreditation and Quality Improvement Program (MBSAQIP): Optimal resources for metabolic and bariatric surgery. https://www.facs.org/-/media/files/quality-programs/bariatric/2019_mbsaqip_standards_manual.ashx (2019). Accessed 7 Aug 2021

[63] Templin T, Hashiguchi TCO, Thomson B, Dieleman J, Bendavid E (2019). The overweight and obesity transition from the wealthy to the poor in low- and middle-income countries: a survey of household data from 103 countries. PLOS Med..

[64] Wallenius V (2020). Sleeve gastrectomy and Roux-en-Y gastric bypass in the treatment of type 2 diabetes. Two-year results from a Swedish multicenter randomized controlled trial, (in eng). Surg. Obes. Relat. Dis..

[65] Yang J (2015). Long-term effects of laparoscopic sleeve gastrectomy versus roux-en-Y gastric bypass for the treatment of Chinese type 2 diabetes mellitus patients with body mass index 28–35 kg/m(2), (in eng). BMC Surg..

[66] Schauer PR (2017). Bariatric surgery versus intensive medical therapy for diabetes—5-year outcomes, (in eng). N. Engl. J. Med..

[67] Mahawar KK (2018). The first consensus statement on one anastomosis/mini gastric bypass (OAGB/MGB) using a modified delphi approach, (in eng). Obes. Surg..

[68] O'Neill KN, Finucane FM, le Roux CW, Fitzgerald AP, Kearney PM (2017). Unmet need for bariatric surgery, (in eng). Surg. Obes. Relat. Dis..

[69] Desogus D, Menon V, Singhal R, Oyebode O (2019). An examination of Who is eligible and who is receiving bariatric surgery in England: secondary analysis of the health survey for England dataset. Obes. Surg..

[70] Dai H, Alsalhe TA, Chalghaf N, Riccò M, Bragazzi NL, Wu J (2020). The global burden of disease attributable to high body mass index in 195 countries and territories, 1990–2017: an analysis of the Global Burden of Disease Study, (in eng). PLoS Med..

[71] Panunzi S, De Gaetano A, Carnicelli A, Mingrone G (2015). Predictors of remission of diabetes mellitus in severely obese individuals undergoing bariatric surgery: do BMI or procedure choice matter? A meta-analysis, (in eng). Ann. Surg..

[72] Rubino F (2020). Bariatric and metabolic surgery during and after the COVID-19 pandemic: DSS recommendations for management of surgical candidates and postoperative patients and prioritisation of access to surgery. Lancet Diabetes Endocrinol..

[73] Squires H, Chilcott J, Akehurst R, Burr J, Kelly MP (2016). A systematic literature review of the key challenges for developing the structure of public health economic models. Int. J. Public Health.

[74] Afzali HHA, Bojke L, Karnon J (2018). Model structuring for economic evaluations of new health technologies. Pharmacoeconomics.

[75] Sjöström L (2004). Lifestyle, diabetes, and cardiovascular risk factors 10 years after bariatric surgery, (in eng). N. Engl. J. Med..

[76] Sjöström L (2014). Association of bariatric surgery with long-term remission of type 2 diabetes and with microvascular and macrovascular complications, (in eng). JAMA.

[77] Dixon JB (2008). Adjustable gastric banding and conventional therapy for type 2 diabetes: a randomized controlled trial, (in eng). JAMA.

[78] Wharton S (2020). Obesity in adults: a clinical practice guideline. Can. Med. Assoc. J..

[79] Einarson TR, Acs A, Ludwig C, Panton UH (2018). Economic burden of cardiovascular disease in type 2 diabetes: a systematic review, (in eng). Value Health.

[80] Cresci B, Cosentino C, Monami M, Mannucci E (2020). Metabolic surgery for the treatment of type 2 diabetes: a network meta-analysis of randomized controlled trials, (in eng). Diabetes Obes. Metab..

[81] Courcoulas AP (2014). Surgical vs medical treatments for type 2 diabetes mellitus: a randomized clinical trial, (in eng). JAMA Surg..

[82] Beaudet A, Clegg J, Thuresson PO, Lloyd A, McEwan P (2014). Review of utility values for economic modeling in type 2 diabetes, (in eng). Value Health.

[83] Dennett SL, Boye KS, Yurgin NR (2008). The impact of body weight on patient utilities with or without type 2 diabetes: a review of the medical literature, (in eng). Value Health.

[84] Xia Q (2020). Health state utilities for economic evaluation of bariatric surgery: a comprehensive systematic review and meta-analysis, (in eng). Obes. Rev..

[85] Ridderstråle M (2016). Estimating the impact of changes in HbA1c, body weight and insulin injection regimen on health related quality-of-life: a time trade off study, (in eng). Health Qual. Life Outcomes.

[86] Bucknor A, Ekwobi C (2014). Need for guidelines on body recontouring after bariatric surgery. BMJ Br. Med. J..

[87] Azin A, Zhou C, Jackson T, Cassin S, Sockalingam S, Hawa R (2014). Body contouring surgery after bariatric surgery: a study of cost as a barrier and impact on psychological well-being, (in eng). Plast. Reconstr. Surg..

[88] Galvain T, Patel S, Kabiri M, Tien S, Casali G, Pournaras DJ (2021). Cost-effectiveness of bariatric and metabolic surgery, and implications of COVID-19 in the United Kingdom, (in eng). Surg. Obes. Relat. Dis..

