# Supplementary material for: Cost-effectiveness of metabolic surgery for the treatment of type 2 diabetes and obesity: a systematic review of economic evaluations
Source: Eur J Health Econ. 2022 Jul 22;24(4):575–90. doi: 10.1007/s10198-022-01494-2 (PMC10175448; doi:10.1007/s10198-022-01494-2)
Supplement: Supplementary file 1 — Supplementary file1 (DOCX 156 kb) [file 10198_2022_1494_MOESM1_ESM.docx]

**Contents**

[**Supplementary Information 1:** Search strategy 2](#_Toc105259216)

[**Supplementary Information 2:** Characteristics of included studies 6](#_Toc105259217)

[**Supplementary Information 3:** Additional results 13](#_Toc105259218)

[**Supplementary Information 4:** Transferability assessment 21](#_Toc105259219)

# **Supplementary Information**

## **Supplementary Information 1: Search strategy**

**Table S1 Research question and inclusion criteria in the PICO framework**

| What is the cost-effectiveness of metabolic surgery for the management of T2D in patients with obesity? | |
| --- | --- |
| Population | Adults ≥ 18 years of age with type 2 diabetes and obesity^a^   1. Patients with obesity and T2D 2. Patients with obesity, where a subgroup of patients have T2DM |
| Intervention | Bariatric/metabolic surgery procedures in current use, performed either as open or laparoscopic procedures |
| Comparator | Non‐surgical management (usual care)^b^ |
| Outcomes | ICER or NMB |
| Study Designs | CUA, CEA |

**Key:** BMI – Body mass index; CEA – cost-effectiveness analysis; CUA – cost-utility analysis; ICER – incremental cost-effectiveness ratio; LYG – life years gained; NMB – net monetary benefit; QALYs – quality-adjusted life years; T2D – Type 2 Diabetes.

^a^ BMI thresholds were not pre-specified as thresholds associated with obesity and obesity-related disease may vary dependent on the target population.

^b^ Usual care can include descriptions such as conservative treatment, conventional or intensive medical management.

The following exclusion criteria were applied:

- commentaries, letters or conference abstracts where a detailed description of the methods was not available
- cost-consequence analysis, cost-benefit analysis, other types of cost analyses or comparative resource use studies
- partial economic evaluations, defined as evaluations that do not assess both costs and consequences and/or do not compare at least two alternatives
- economic evaluations of metabolic surgery for the prevention of T2D
- Studies that were not available in English.

**Table S2: Medline (Ovid) search strategy**

| # | Query |
| --- | --- |
| 1 | exp Diabetes Mellitus/ |
| 2 | (Diabetes or diabetes mellitus or diabetic or Type 2 diabetes mellitus or type 2 diabetes or T2D or T2DM or non-insulin-dependent diabetes).mp. |
| 3 | (Diabetes complications or diabetic complications or microvascular complications or macrovascular complications or retinopathy or neuropathy or nephropathy).mp. |
| 4 | (obesity-related comorbidit* or obesity-related co-morbidit* or obesity-associated comorbidit* or obesity-associated co-morbidit* or obesity-related disease* or obesity-associated disease* or obesity-related complication* or obesity-associated complication* or obesity-related condition* or obesity-associated condition* or obesity-related adverse outcome* or obesity-related adverse event* or co-morbid* or comorbid*).mp. |
| 5 | Comorbidity/ |
| 6 | 1 or 2 or 3 or 4 or 5 |
| 7 | exp Bariatric surgery/ |
| 8 | Bariatric surgery.mp. |
| 9 | metabolic surgery.mp. |
| 10 | weight loss surgery.mp. |
| 11 | obesity surgery.mp. |
| 12 | (Roux-en-Y or RYGB).mp. |
| 13 | (gastric bypass or gastrojejunal bypass or gastro-jejunal bypass or gastroileal bypass or gastro-ileal bypass or duodenojejunal bypass or duodeno-jejunal bypass or duodenoileal bypass).mp. |
| 14 | (mini gastric bypass or MGB or one anastomosis gastric bypass or OAGB or single anastomosis gastric bypass or SAGB or omega loop gastric bypass).mp. |
| 15 | (sleeve gastrectomy or gastric sleeve or VSG or SG).mp. |
| 16 | (gastric band* or intragastric band* or gastroplast* or vertical band or lapband or lap-band or adjustable band or AGB).mp. |
| 17 | (biliopancreatic diversion or bilio-pancreatic diversion or duodenal switch or BPD-DS).mp. |
| 18 | (Single Anastomosis Duodeno-Ileal Bypass with Sleeve Gastrectomy or SADI-S or SADIS).mp. |
| 19 | (Single Anastomosis Sleeve Ileal Bypass with Sleeve Gastrectomy or SASI).mp. |
| 20 | 7 or 8 or 9 or 10 or 11 or 12 or 13 or 14 or 15 or 16 or 17 or 18 or 19 |
| 21 | Economics/ |
| 22 | "costs and cost analysis"/ |
| 23 | Cost allocation/ |
| 24 | Cost-benefit analysis/ |
| 25 | Cost control/ |
| 26 | Cost savings/ |
| 27 | Cost of illness/ |
| 28 | Cost sharing/ |
| 29 | "deductibles and coinsurance"/ |
| 30 | Medical savings accounts/ |
| 31 | Health care costs/ |
| 32 | Direct service costs/ |
| 33 | Drug costs/ |
| 34 | Employer health costs/ |
| 35 | Hospital costs/ |
| 36 | Health expenditures/ |
| 37 | Capital expenditures/ |
| 38 | Value of life/ |
| 39 | exp economics, hospital/ |
| 40 | exp economics, medical/ |
| 41 | Economics, nursing/ |
| 42 | Economics, pharmaceutical/ |
| 43 | exp "fees and charges"/ |
| 44 | exp budgets/ |
| 45 | (low adj cost).mp. |
| 46 | (high adj cost).mp. |
| 47 | (health?care adj cost$).mp. |
| 48 | (fiscal or funding or financial or finance).tw. |
| 49 | (cost adj estimate$).mp. |
| 50 | (cost adj variable).mp. |
| 51 | (unit adj cost$).mp. |
| 52 | (economic$ or pharmacoeconomic$ or price$ or pricing).tw. |
| 53 | 21 or 22 or 23 or 24 or 25 or 26 or 27 or 28 or 29 or 30 or 31 or 32 or 33 or 34 or 35 or 36 or 37 or 38 or 39 or 40 or 41 or 42 or 43 or 44 or 45 or 46 or 47 or 48 or 49 or 50 or 51 or 52 |
| 54 | 6 and 20 and 53 |

**Table S3: Embase search strategy**

| No. | Query |
| --- | --- |
| #1 | 'diabetes mellitus'/exp |
| #2 | diabetes:ab,ti OR 'diabetes mellitus':ab,ti OR diabetic:ab,ti OR 'type 2 diabetes mellitus':ab,ti OR 'type 2 diabetes':ab,ti OR t2d:ab,ti OR t2dm:ab,ti OR 'non-insulin-dependent diabetes':ab,ti |
| #3 | 'diabetes complications':ab,ti OR 'diabetic complication*':ab,ti OR 'microvascular complication*':ab,ti OR 'microvascular disease*':ab,ti OR 'macrovascular complication*':ab,ti OR 'macrovascular disease*':ab,ti OR retinopathy:ab,ti OR neuropathy:ab,ti OR nephropathy:ab,ti |
| #4 | 'obesity-related comorbidit*':ab,ti OR 'obesity-related co-morbidit*':ab,ti OR 'obesity-associated comorbidit*':ab,ti OR 'obesity-associated co-morbidit*':ab,ti OR 'obesity-related disease*':ab,ti OR 'obesity-associated disease*':ab,ti OR 'obesity-related complication*':ab,ti OR 'obesity-associated complication*':ab,ti OR 'obesity-related condition*':ab,ti OR 'obesity-associated condition*':ab,ti OR 'obesity-related adverse outcome*':ab,ti OR 'obesity-related adverse event*':ab,ti OR 'co-morbid*':ab,ti OR 'comorbid*':ab,ti OR 'comorbidity'/exp |
| #5 | OR 1-4 |
| #6 | 'bariatric surgery'/exp |
| #7 | 'bariatric surgery':ab,ti |
| #8 | 'metabolic surgery':ab,ti |
| #9 | 'weight loss surgery':ab,ti |
| #10 | 'obesity surgery':ab,ti |
| #11 | 'roux-en-y gastric bypass'/exp OR 'roux-en-y':ab,ti OR rygb:ab,ti |
| #12 | 'gastric bypass surgery'/exp OR 'gastric bypass':ab,ti OR 'gastrojejunal bypass':ab,ti OR 'gastro-jejunal bypass':ab,ti OR 'gastroileal bypass':ab,ti OR 'gastro-ileal bypass':ab,ti OR 'duodenojejunal bypass':ab,ti OR 'duodeno-jejunal bypass':ab,ti OR 'duodenoileal bypass':ab,ti |
| #13 | 'mini gastric bypass':ab,ti OR mgb:ab,ti OR 'one anastomosis gastric bypass':ab,ti OR oagb:ab,ti OR 'single anastomosis gastric bypass':ab,ti OR sagb OR 'omega loop gastric bypass':ab,ti |
| #14 | 'sleeve gastrectomy'/exp OR 'sleeve gastrectomy':ab,ti OR 'gastric sleeve':ab,ti OR vsg:ab,ti OR sg:ab,ti |
| #15 | 'gastric band':ab,ti OR 'intragastric band':ab,ti OR 'gastroplasty':ab,ti OR 'vertical band':ab,ti OR lapband:ab,ti OR 'lap band':ab,ti OR 'adjustable band':ab,ti OR agb:ab,ti |
| #16 | 'biliopancreatic bypass'/exp OR 'biliopancreatic diversion':ab,ti OR 'bilio-pancreatic diversion':ab,ti OR 'duodenal switch':ab,ti OR 'bpd ds':ab,ti OR bpd:ab,ti |
| #17 | 'single anastomosis duodeno–ileal bypass with sleeve gastrectomy':ab,ti OR 'sadi s':ab,ti OR sadis:ab,ti |
| #18 | 'single anastomosis sleeve ileal bypass with sleeve gastrectomy':ab,ti OR sasi:ab,ti |
| #19 | OR 6-18 |
| #20 | 'socioeconomics'/exp |
| #21 | 'cost benefit analysis'/exp |
| #22 | 'cost effectiveness analysis'/exp |
| #23 | 'cost of illness'/exp |
| #24 | 'cost control'/exp |
| #25 | 'economic aspect'/exp |
| #26 | 'financial management'/exp |
| #27 | 'health care cost'/exp |
| #28 | 'health care financing'/exp |
| #29 | 'health economics'/exp |
| #30 | 'hospital cost'/exp |
| #31 | fiscal:ti,ab,de OR financial:ti,ab,de OR finance:ti,ab,de OR funding:ti,ab,de |
| #32 | 'cost minimization analysis'/exp |
| #33 | cost NEXT/1 estimate$ |
| #34 | cost NEXT/1 variable$ |
| #35 | unit NEXT/1 cost$ |
| #36 | OR 20-36 |
| #37 | #5 AND #19 AND #36 |

## **Supplementary Information 2: Characteristics of included studies**

**Table S4 Characteristics of included studies**

| Author (year) | Target population | Measure of treatment effect | Intervention | Comparator |
| --- | --- | --- | --- | --- |
| MODEL-BASED ECONOMIC EVALUATIONS | | | | |
| T2DM population or sub-cohort | | | | |
| Ackroyd  (2006) | BMI ≥35kg/m^2^ and T2D, after failure of at least 1 year of medical treatment | T2D-free years; BMI year (combines magnitude and duration of BMI variation) | AGB; GBP | Usual diabetes care |
| Anselmino  (2009) | BMI ≥35 kg/m^2^ after failure of at least 1 year of medical treatment | See Ackroyd (2006) | AGB; GBP | Usual diabetes care |
| Assumpção  (2019) | Severely obese individuals (BMI >35 kg/m^2^) with and without T2D | T2D remission; Fatal and non-fatal MI | RYGB (open) | Usual diabetes care |
| Gil-Rojas  (2019) | 1. BMI ≥40 kg/m^2^ with or without comorbidities, 2. BMI 35 - 40 kg/m^2^ with comorbidities (T2D, sleep apnoea, hypertension or dyslipidaemia) | Remission of T2D; weight reduction (Risk of stroke and AMI linked to BMI) | GBP; SG | Pharmacologic treatment (orlistat) and lifestyle changes (diet and exercise) |
| Hoerger  (2010) | Severely obese (BMI ≥35 kg/m^2^) adults with newly-diagnosed (<5 years) or established diabetes (>10 years) | T2D remission; SBP; total cholesterol; HDL; BMI | AGB; GBP | Usual diabetes care |
| Ikramuddin  (2009) | BMI ≥35 kg/m^2^ and T2D after failure of  1 year of medical treatment | SBP, BMI, HbA1C, lipid parameters (total cholesterol, LDL-cholerterol, HDL cholesterol, triglycerides) | RYGB | Usual diabetes care |
| Keating  (2009b) | Recently diagnosed T2D in class I/II obesity | Number of years in T2D remission | LAGB | Usual diabetes care |
| Kim  (2018) | 10 subgroups based on 5 levels of BMI (30–34.9; 35–39.9; 40–44.9; 45–49.9; >50) with or without T2D | BMI values and self-reported T2D status | LRYGB (base case); ORYGB; LAGB | Non-surgical intervention |
| McGlone  (2020) | Insulin-dependent T2D | BMI; HbA1c; SBP and total cholesterol to HDL ratio | RYGB (58%) procedure); SG (42%) | best medical treatment (including nutritional counselling)  Year 1: Insulin + Metformin + DPP4 inhibitors  Year 2: Insulin + Metformin + GLP-1 RA  Year 3: Insulin + Metformin + SGLT2 inhibitor + GLP-1 RA  Year 4: Insulin + Metformin + SGLT2 inhibitor + GLP-1 RA  Year 5: Insulin + Metformin + SGLT2 inhibitor + GLP-1 RA |
| Pollock  (2013) | Obese patients with T2D | BMI, HbA1c, SBP, lipid parameters (total cholesterol; triglycerides; HDL); minor and hypoglycaemic events | LAGB | Usual diabetes care |
| Rognoni  (2020) | 1. BMI 40 kg/m^2^ without complications + patients with BMI 35 kg/m^2^ with complications; 2. BMI 35 kg/m^2^ and T2D; 3. BMI 30 to 35 kg/ m^2^ and T2D | SBP, lipids, T2D (dependent on BMI) | AGB (16.8%); GBP(24.6%); SG (58.6%) | Usual diabetes care |
| Tang  (2016) | 1. Aged 16 to 65 years; 2. BMI ≥28 kg/m^2^; 3. T2D (≤15 years duration) | partial remission; complete remission | LSG; LRYGB | Usual diabetes care (metformin, sulfonylurea, and insulin) |
| Viratanapanu  (2019) | T2D with BMI >32.5 kg/m^2^ | BMI and HbA1c | RYGB (61.6%) | Usual diabetes care (Metformin, Sulfonylurea group, Thiazolidinedione group, Alpha-glucosidase inhibitors group, Insulin) |
| Wan  (2019) | 1. Aged 18 to 65 years with recently diagnosed T2D (within 2 years) 2. BMI >28kg/m^2^ 3. Fasting serum c-peptide in the lower 1/2 of the lower limits of normal | Remission of T2D | LRYGB | Usual diabetes care (metformin, sulfonylurea, and insulin) |
| Population subgroup with T2D | | | | |
| Borisenko (2018)a | Subgroup analyses were performed for 8 diabetic cohorts, namely, males and females with:   1. moderate obesity (BMI 33 kg/m^2^), 2. severe obesity (BMI 37 kg/m^2^), 3. morbid obesity (BMI 42 kg/m^2^) 4. super obesity (BMI 52 kg/m^2^) | BMI, SBP and T2D.  Risk of obesity-related CVD is dependent on patient characteristics: age, sex, SBP, BMI, T2D and smoking status. | GBP (56%);  SG (22%);  AGB (22%); | Usual diabetes care |
| Borisenko (2018)b |  |  | GBP (75%);  SG (20%);  AGB (5%) |  |
| Borisenko (2017)a |  |  | GBP (68.8%);  SG (31%);  AGB (0.2%) |  |
| Borisenko (2017)b |  |  | GBP (51%);  SG (17%);  AGB (33%) |  |
| Borisenko (2015) |  |  | GBP (98 %);  SG (1.6 %,);  AGB (0.4 %) |  |
| Cohen (2017) | 1. BMI ≥40 kg/m^2^ 2. BMI ≥35 kg/m^2^ with co-morbidities | BMI, T2D, SBP, and lipid profile | Open RYGB (90%) | Usual diabetes care |
| Faria (2013) | Patients with T2D and overweight (BMI 25-30 kg/m^2^) or obesity (BMI ≥30 kg/m^2^) | Weight loss (associated with comorbidity improvement) | GB; GBP | Usual diabetes care |
| Gulliford (2017) | Morbid obesity (BMI ≥40 kg/m^2^) and diabetes | Remission of T2D | AGB (33%); GBP (33%); SG (33%) | Usual diabetes care |
| James (2017) | Diabetes and obesity (BMI >35 kg/m^2^) | BMI | AGB; RYGB; SG | Usual diabetes care  (including orlistat) |
| Klarenbach (2010) | 1. BMI of 40 kg/m^2^ or more 2. BMI of 35 kg/m^2^ or more with a major obesity-related comorbidity | BMI; prevalence of obesity-related comorbidities | RYGB (base case); LAGB; SG; BPD | Usual diabetes care |
| Lucchese (2017) | Subgroup analyses were performed for 8 diabetic cohorts, namely, males and females with:   1. moderate obesity (BMI 33kg/m^2^), 2. severe obesity (BMI 37kg/m^2^), 3. morbid obesity (BMI 42kg/m^2^) 4. super obesity (BMI 52kg/m^2^) | BMI, SBP, and T2D | GBP; SG; AGB | Usual diabetes care |
| McEwen (2010) | 1. BMI 35 kg/m^2^ with two life threatening comorbidities or 2. 40 kg/m^2^ | BMI, comorbidity improvement | ORYGB (64%)  LRYGB (33%) | Usual diabetes care |
| Picot (2012) | Class I and class II obesity (BMI ≥30 and <40), with T2D | BMI; prevalence of obesity-related comorbidities | LAGB | Usual diabetes care |
| Sanchez-Santos (2017) | Subgroup analyses were performed for 8 diabetic cohorts, namely, males and females with:   1. moderate obesity (BMI 33kg/m^2^), 2. severe obesity (BMI 37kg/m^2^), 3. morbid obesity (BMI 42kg/m^2^) 4. super obesity (BMI 52kg/m^2^) | BMI, SBP, and T2D | GBP (76%); SG (22%); AGB (2%) | Usual diabetes care |
| EMPIRICAL EVIDENCE-BASED ECONOMIC EVALUATIONS | | | | |
| T2D population | | | | |
| Keating  (2009a) | Recently diagnosed T2D (<2 years) with class I/II obesity | Cases of T2D remitted | LAGB | Usual diabetes care |
| Tu  (2019) | 1. Poorly controlled T2D (duration ≤15 years) with adequate islet function 2. age 18–65 years; 3. BMI ≥27.5 kg/m^2^; 4. >2 symptoms of the metabolic syndrome | HbA1c values | LRYGB | Usual diabetes care |

**Key:** AGB – adjustable gastric band; BMI – body mass index; BPD – biliopancreatic diversion; CVD – cardiovascular disease; GB- gastric band; GBP - gastric bypass (generally RYGB); HbA1c – Haemoglobin A1c; HDL – high-density lipoprotein; LAGB – laparoscopic adjustable gastric band; LDL – low-density lipoprotein; LRYGB – laparoscopic Roux-en-Y gastric bypass; LSG – laparoscopic sleeve gastrectomy; ORYGB – open Roux-en-Y Gastric Bypass; QoL – quality of life; RYGB - Roux-en-Y Gastric Bypass; SBP – systolic blood pressure; SG – sleeve gastrectomy; T2D type 2 diabetes.

**Table S5 Diabetes-related and surgical complications included in model-based studies^a^**

|  | T2D status | | Macrovascular complications | | | | | | Microvascular complications | | |  |  |  |
| --- | --- | --- | --- | --- | --- | --- | --- | --- | --- | --- | --- | --- | --- | --- |
| Author (year) | T2D remission | T2D improvement | Stroke | CHD | MI | Angina | HF | PAD | Nephropathy or  ESRD | Neuropathy | Retinopathy or blindness | Foot ulcer or amputation | Acute glycaemic event | Surgical complications |
| T2D population or sub-cohort | | | | | | | | | | | | | | |
| Ackroyd (2006) | x |  |  |  |  |  |  |  |  |  |  |  |  |  |
| Anselmino (2009) | x |  |  |  |  |  |  |  |  |  |  |  |  |  |
| Assumpção (2019) | x |  |  |  | x |  |  |  |  |  |  |  |  | x^b^ |
| Gil-Rojas (2019) | x |  | x |  | x |  |  |  |  |  |  |  |  | x |
| Hoerger (2010) | x | x | x | x |  |  |  |  | x | x | x |  |  |  |
| Ikramuddin (2009) | x | x^c^ | x |  | x | x | x | x | x | x | x | x | x | x |
| Keating (2009b) | x |  |  |  |  |  |  |  |  |  |  |  |  |  |
| Kim (2018) | x |  |  |  |  |  |  |  |  |  |  |  |  |  |
| McGlone (2020) | x | x^c^ | x | x | x |  | x |  | x |  | x | x |  | x |
| Pollock (2013) | x | x^c^ | x |  | x | x | x | x | x | x | x | x | x |  |
| Rognoni (2020) | x |  | x |  | x |  |  |  | x |  | x | x | x | x |
| Tang (2016) | x |  |  |  |  |  |  |  |  |  |  |  |  |  |
| Viratanapanu (2019) | x | x |  |  |  |  |  |  |  |  |  |  |  |  |
| Wan (2019) | x |  |  |  |  |  |  |  |  |  |  |  |  |  |
| T2D subpopulation | | | | | | | | | | | | | | |
| Borisenko (2018)a | x |  | x |  | x | x | x | x |  |  |  |  |  | x^b^ |
| Borisenko (2018)b | x |  | x |  | x | x | x | x |  |  |  |  |  | x^b^ |
| Borisenko (2017)a | x |  | x |  | x | x | x | x |  |  |  |  |  | x^b^ |
| Borisenko (2017)b | x |  | x |  | x | x | x | x |  |  |  |  |  | x^b^ |
| Borisenko (2015) | x |  | x |  | x | x | x | x |  |  |  |  |  | x^b^ |
| Cohen (2017) | x |  | x | x |  |  | x |  |  |  |  |  |  | x |
| Faria (2013) ^d,e^ |  |  | x | x | x | x | x |  | x |  |  |  |  | x^b^ |
| Gulliford (2017) | x |  | x | x |  |  |  |  |  |  |  |  |  | x |
| James (2017)^e^ |  |  |  |  |  |  |  |  |  |  |  |  |  |  |
| Klarenbach (2010) | x |  |  |  |  |  |  |  |  |  |  |  |  | x |
| Lucchese (2017) | x |  | x |  | x | x | x | x |  |  |  |  |  | x^b^ |
| McEwen (2010)^e^ |  |  |  |  |  |  |  |  |  |  |  |  |  |  |
| Picot (2012) | x |  | x | x |  |  |  |  |  |  |  |  |  |  |
| Sanchez-Santos (2017) | x |  | x |  | x | x | x | x |  |  |  |  |  | x^b^ |

**Key:** CHD – coronary heart disease/ischemic heart disease/coronary artery disease; ESRD – end-stage renal disease; HF – heart failure; MI – myocardial infraction; PAD – peripheral arterial disease; T2D – Type 2 diabetes.

^a^ While some models incorporated other obesity-related health states (for example, sleep apnoea, cancer), only T2D-related health states are presented. Economic evaluations based on a trial or observational study (empirical evidence-based economic evaluations) are not presented.

^b^ No disutility assigned to surgical complications health state.

^c^ Treatment effect based on change in HbA1c.

^d^ Specific cardiovascular health states not reported. Inclusion of all relevant cardiovascular events is assumed.

^e^ Adaptation of the model structure to include T2D-related health states was not reported.

## **Supplementary Information 3: Additional results**

**Interpretation of cost-effectiveness**

Context-specific ICERs and willingness-to-pay (WTP) thresholds adopted in the reference country or by the study authors where no established WTP threshold exists are presented in Table S6. ICERs were adjusted to a common cost year (2020) and currency (Euro) using consumer price indices (CPIs) and purchasing power parities (PPPs) to facilitate comparison across studies and interpretation in the Irish context. In Ireland, WTP thresholds of €20,000 and €45,000 per quality-adjusted life year (QALY) gained are commonly employed to aid interpretation of cost-effectiveness analyses.^(1)^ ICERs can be interpreted as follows: <€20,000/QALY – cost-effective; €20-45,000/QALY – possibly cost-effective; >€45,000/QALY – not cost-effective. WTP thresholds adopted in Ireland are broadly consistent with thresholds commonly adopted in other European countries.^(2, 3)^

In general, interpretation of the evidence in the Irish context was consistent with the findings of the original study (Table S6). In two studies, the ICER marginally exceeded the WTP threshold of €20,000/QALY, but would still be considered cost-effective at a threshold of €45,000/QALY.^(4, 5)^

**Table S6 Cost-effectiveness of metabolic surgery in the original and Irish context**

| **Author (year)** | **Country** | **Cost year** | **Original context** | | | **Irish context** | |
| --- | --- | --- | --- | --- | --- | --- | --- |
|  |  |  | **Unadjusted ICER** | **WTP threshold** | **Interpretation** | **Adjusted ICER^a,b^ (€/QALY or €/case of T2D remitted)** | **Interpretation at WTP thresholds of €20,000 and €45,000 per QALY** |
| **MODEL-BASED ECONOMIC EVALUATIONS** | | | | | | | |
| **T2D population or sub-cohort** | | | | | | | |
| *Bariatric surgery versus usual care^c^* | | | | | | | |
| Gil-Rojas (2019) | Columbia | 2016 | 6,194,899 COP | COP $16,613,951  (cost-effective)  or  $16,613,951 -49,841,853 (potentially cost-effective) | Cost-effective | 4,531/QALY | Cost-effective |
| McGlone (2020) | United Kingdom | 2018 | Dominant | GBP £20,000/QALY | Cost-saving | Dominant | Cost-saving |
| Rognoni (2020) | Italy | 2018 | Dominant | €30,000/QALY | Cost-saving | Dominant | Cost-saving |
| *Gastric banding versus usual care* | | | | | | | |
| Ackroyd (2006) | Germany | 2005 | Dominant | Not reported | Cost-saving | Dominant | Cost-saving |
|  | France | 2005 | Dominant | Not reported | Cost-saving | Dominant | Cost-saving |
|  | United Kingdom | 2005 | £1,929 | GBP £20,000/QALY | Cost-effective | 3,269/QALY | Cost-effective |
| Anselmino (2009) | Austria | 2009 | Dominant | Not reported | Cost-saving | Dominant | Cost-saving |
|  | Italy | 2009 | Dominant | Not reported | Cost-saving | Dominant | Cost-saving |
|  | Spain | 2009 | €1,456 | €30,000/QALY | Cost-effective | 2,104/QALY | Cost-effective |
| Hoerger (2010) | United States | 2005 | USD $ 12,098^d^ | USD $50,000/QALY | Cost-effective | 15,848/QALY | Cost-effective |
| Keating (2009)b | Australia | 2006 | Dominant | AUD $50,000/QALY | Cost-saving | Dominant | Cost-saving |
| Kim (2018) | United States | 2014 | US $7,789^d^ | USD $100,000/QALY | Cost-effective | 7,577/QALY | Cost-effective |
| Pollock (2013) | United Kingdom | 2010 | £3,602 | GBP £50,000/QALY | Cost-effective | 5,275/QALY | Cost-effective |
| *Gastric bypass versus usual care* | | | | | | | |
| Ackroyd (2006) | Germany | 2005 | Dominant | Not reported | Cost-saving | Dominant | Cost-saving |
|  | France | 2005 | Dominant | Not reported | Cost-saving | Dominant | Cost-saving |
|  | United Kingdom | 2005 | £1,517 | GBP £20,000/QALY | Cost-effective | 2,571/QALY | Cost-effective |
| Anselmino (2009) | Austria | 2009 | Dominant | Not reported | Cost-saving | Dominant | Cost-saving |
|  | Italy | 2009 | Dominant | Not reported | Cost-saving | Dominant | Cost-saving |
|  | Spain | 2009 | €2,664 | €30,000/QALY | Cost-effective | 3,850/QALY | Cost-effective |
| Assumpção (2019) | Brazil | 2015 | Int $1,820 | Int $15,565/QALY | Cost-effective | 1,278/QALY | Cost-effective |
| Hoerger (2010) | United States | 2005 | USD $9,172^d^ | USD $50,000/QALY | Cost-effective | 12,015/QALY | Cost-effective |
| Ikramuddin (2009) | United States | 2007 | US $21,973 | USD $50,000/QALY | Cost-effective | 26,502/QALY | Possibly cost-effective |
| Kim (2018) | United States | 2014 | $7,844^d,e^ | USD $100,000/QALY | Cost-effective | 7,630/QALY | Cost-effective |
| Tang (2016) | China | Not reported^f^ | Int $451^g^ | Not reported | Cost-effective^h^ | 116/QALY | Cost-effective |
| Viratanapanu (2019) | Thailand | 2017 | 26,908 THB | THB 150-200,000/QALY | Cost-effective | 1,863/QALY | Cost-effective |
| Wan (2019) | China | 2015 | Dominant | ¥193,932/QALY | Cost-saving | Dominant | Cost-saving |
| *Sleeve gastrectomy versus usual care* | | | | | | | |
| Tang (2016) | China | Not reported^f^ | Int $361^g^ | Not reported | Cost-effective^h^ | 92/QALY | Cost-effective |
| **T2D subpopulation** | | | | | | | |
| *Bariatric surgery versus usual care^c^* | | | | | | | |
| Borisenko (2018)a | England | 2015 | Dominant | GBP £30,000/QALY | Cost-saving | Dominant | Cost-saving |
| Borisenko (2018)b | Belgium | 2012 | Dominant | €30,000/QALY | Cost-saving | Dominant | Cost-saving |
| Borisenko (2017)a | Denmark | 2012 | Dominant | DKK 223-250,000/QALY | Cost-saving | Dominant | Cost-saving |
| Borisenko (2017)b | Germany | 2012 | Dominant | €35,000/QALY | Cost-saving | Dominant | Cost-saving |
| Borisenko (2015) | Sweden | 2012 | Dominant | €35,526/QALY | Cost-saving | Dominant | Cost-saving |
| Gulliford (2017) | United Kingdom | 2013 | £6,176 | GBP £30,000/QALY | Cost-effective | 8,296/QALY | Cost-effective |
| Lucchese(2017) | Italy | 2013 | Dominant | €50,000/QALY | Cost-saving | Dominant | Cost-saving |
| Sanchez-Santos (2017) | Spain | 2017 | Dominant | €30,000/QALY | Cost-saving | Dominant | Cost-saving |
| *Gastric banding versus usual care* | | | | | | | |
| Faria (2013) | Portugal | Not reported^f^ | €1,810^d^ | €20,000/QALY | Cost-effective | 2,845/QALY | Cost-effective |
| James (2017) | Australia | 2015 | Dominant | AUD $70,000/QALY | Cost-saving | Dominant | Cost-saving |
| Picot (2012) | United Kingdom | 2009 | US $8,831 |  |  | 2,462/QALY | Cost-effective |
| *Gastric bypass versus usual care* | | | | | | | |
| Cohen (2017) | Brazil | Not reported^f^ | Dominant | Int $16,500 | Cost-saving | Dominant | Cost-saving |
| Faria (2013) | Portugal | Not reported^f^ | Dominant | €20,000/QALY | Cost-saving | Dominant | Cost-saving |
| James (2017) | Australia | 2015 | Dominant | AUD $70,000/QALY | Cost-saving | Dominant | Cost-saving |
| Klarenbach (2010) | Australia | 2009 | Dominant | $20,000 and $50,000/QALY | Cost-saving | Dominant | Cost-saving |
| McEwen (2010) | United States | 2007 | USD $8,831 | Not reported | Cost-effective^i^ | 10,651/QALY | Cost-effective |
| *Sleeve gastrectomy versus usual care* | | | | | | | |
| James (2017) | Australia | 2015 | Dominant | AUD $70,000/QALY | Cost-saving | Dominant | Cost-saving |
| **EMPIRICAL EVIDENCE-BASED ECONOMIC EVALUATIONS** | | | | | | | |
| **T2D population** | | | | | | | |
| *Gastric banding versus usual care* | | | | | | | |
| Keating (2009)a | Australia | 2006 | 16,600 | AUD $50,000/QALY | Cost-effective | 16,554/case of T2D remitted | Cost-effective |
| *Gastric bypass versus usual care* | | | | | | | |
| Tu (2019) | China | 2013 | ¥ 125,836  (USD $19,359) | $20,277/QALY | Cost-effective | 32,270/QALY | Possibly cost-effective |

**Key:** AUD – Australian dollars; COP – Colombian pesos; DKK – Danish Krone; GBP – Great British pound (Pound sterling); ICER – incremental cost effectiveness ratio; Int $ - international dollar; QALY – quality-adjusted life year; THB - Thai baht; USD – United States dollars.

^a^ Where multiple time horizons were used, results for the longest time horizon are presented.

^b^ Adjusted ICER is defined as inflation of a context-specific ICER using country-specific consumer price indices (CPI) to a common cost year (2020), prior to conversion to a common currency (Irish Euro) using purchasing power parities (PPPs). PPPs are indicators of price level differences between countries. Even in countries using a common currency (e.g., Euro), differences in local economies influence the price of products.

^c^ Bariatric surgery comprises a mix of surgeries, typically based on the mix of surgeries in use in clinical practice in the index country.

^d^ Where ICERs were presented by subgroup only, a simple or weighted average ICER was calculated.

^e^ A weighted-average ICER for laparoscopic Roux-en-Y gastric bypass is presented. ICERs for open RYGB are presented in the supplementary information, Table S7.

^f^ Where the cost year was not reported, the average interval between the cost and publication year in other included studies (3 years) was assumed.

^g^ ICERs were not presented in the original study. ICERs were calculated based on the incremental costs and QALYs provided.

^h^ Study author’s interpretation.

**Table S7 Results of CEA or CUA (Additional analyses by time horizon, perspective and/or surgical procedure)**

| Author | Country | Additional analysis | Incremental cost-effectiveness ratio (ICER) (€/QALY) | | | | |
| --- | --- | --- | --- | --- | --- | --- | --- |
| T2D population or sub-cohort | | | | | | | |
| Hoerger (2010) | United States | GBP v usual care; Subgroup (age) | **Newly-diagnosed T2D:**  9,170/QALY | **Established T2D:**  15,720/QALY | **Newly-diagnosed T2D**  **Age 35-44 years:** 6,550/QALY  **Age 65-74 years:** 15,720/QALY | **Established T2D**  **Age 45–54 years:** 11,790/QALY  **Age 65-74 years:** 23,579/QALY |  |
|  |  | GB v usual care; Subgroup (age) | **Newly-diagnosed T2D:**  14,410/QALY | **Established T2D:**  17,029/QALY | **Newly-diagnosed T2D**  **Age 35-44 years:** 11,790/QALY  **Age 65-74 years:** 22,269/QALY | **Established T2D**  **Age 45–54 years:**14,410/QALY  **Age 65-74 years:** 24,889/QALY |  |
| Kim (2018) | United States | LRYGB; 5-year time horizon | **BMI 50 kg/m^2^**  Male: 15,464; Female: 14,817 | **BMI 45 kg/m^2^**  Male: 16,488;  Female: 15,681 | **BMI 40 kg/m^2^**  Male: 17,713; Female: 16,717 | **BMI 35 kg/m^2^**  Male: 19,345; Female: 17,937 | **BMI 30 kg/m^2^**  Male: 20,858; Female: 19,361 |
|  |  | LRYGB; Lifetime horizon | **BMI 50 kg/m^2^**  Male: 5,727  Female: 5,638 | **BMI 45 kg/m^2^**  Male: 6,264  Female: 6,157 | **BMI 40 kg/m^2^**  Male: 6,923  Female: 6,758 | **BMI 35 kg/m^2^**  Male: 7,696  Female: 7,480 | **BMI 30 kg/m^2^**  Male: 8,560  Female: 8,341 |
|  |  | AGB; 5-year time horizon | **BMI 50 kg/m^2^**  Male: 13,437  Female: 12,455 | **BMI 45 kg/m^2^**  Male: 14,296  Female: 13,163 | **BMI 40 kg/m^2^**  Male: 15,333  Female: 13,960 | **BMI 35 kg/m^2^**  Male: 16,476  Female: 14,798 | **BMI 30 kg/m^2^**  Male: 17,827  Female: 16,077 |
|  |  | AGB; Lifetime time horizon | **BMI 50 kg/m^2^**  Male: 6,145  Female: 6,010 | **BMI 45 kg/m^2^**  Male: 6,597  Female: 6,370 | **BMI 40 kg/m^2^**  Male: 7,120  Female: 6,801 | **BMI 35 kg/m^2^**  Male: 7,680  Female: 7,369 | **BMI 30 kg/m^2^**  Male: 8,396  Female: 8,052 |
|  |  | ORYGB; 5-year time horizon | **BMI 50 kg/m^2^**  Male: 28,500  Female: 28,963 | **BMI 45 kg/m^2^**  Male: 29,491  Female: 29,981 | **BMI 40 kg/m^2^**  Male: 31,313  Female: 31,513 | **BMI 35 kg/m^2^**  Male: 33,444  Female: 32,662 | **BMI 30 kg/m^2^**  Male: 35,241  Female: 34,369 |
|  |  | ORYGB; lifetime time horizon | **BMI 50 kg/m^2^**  Male: 10,639  Female: 11,375 | **BMI 45 kg/m^2^**  Male: 11,346  Female: 12,064 | **BMI 40 kg/m^2^**  Male: 12,244  Female: 12,860 | **BMI 35 kg/m^2^**  Male: 13,279  Female: 13,810 | **BMI 30 kg/m^2^**  Male: 14,405  Female: 14,938 |
| Pollock (2013) | United Kingdom | Multiple time horizons | 25,153/QALY (10 years) | 8,681/QALY (20 years) | 5,418/QALY (30 years) | 5,275/QALY (40 years) |  |
| Rognoni (2020) | Italy | Public payer perspective; BMI category | **BMI ≥30-34.9 kg/m^2^**  Dominant | **BMI ≥35 kg/m^2^**  Dominant |  |  |  |
|  |  | Societal perspective; BMI category | **BMI ≥30-34.9 kg/m^2^**  Dominant | **BMI ≥35 kg/m^2^**  Dominant |  |  |  |
| T2D subpopulation | | | | | | | |
| Borisenko (2018)a | England | 10-year time horizon | **BMI 33kg/m^2^**  Male: Dominant; Female: Dominant | **BMI 37 kg/m^2^**  Male: 641/QALY;  Female: Dominant | **BMI 42 kg/m^2^**  Male: 318/QALY; Female: Dominant | **BMI 52 kg/m^2^**  Male: Dominant; Female: Dominant |  |
| Borisenko (2018)b | Belgium | 10-year time horizon | **BMI 33kg/m^2^**  Male: Dominant; Female: Dominant | **BMI 37 kg/m^2^**  Male: Dominant; Female: Dominant | **BMI 42 kg/m^2^**  Male: Dominant; Female: Dominant | **BMI 52 kg/m^2^**  Male: Dominant; Female: Dominant |  |
| Borisenko (2017)a* | Denmark | 10-year time horizon | **BMI 33kg/m^2^**  Male: Dominant; Female: Dominant | **BMI 37 kg/m^2^**  Male: Dominant; Female: Dominant | **BMI 42 kg/m^2^**  Male: Dominant; Female: Dominant | **BMI 52 kg/m^2^**  Male: Dominant; Female: Dominant |  |
| Borisenko (2017)b | Germany | 10-year time horizon | **BMI 33kg/m^2^**  Male: Dominant; Female: Dominant | **BMI 37 kg/m^2^**  Male: Dominant; Female: Dominant | **BMI 42 kg/m^2^**  Male: Dominant; Female: Dominant | **BMI 52 kg/m^2^**  Male: Dominant; Female: Dominant |  |
| Faria | Portugal | GB; lifetime | **BMI 30-35 kg/m^2^**  44,704/QALY | **BMI 35-40 kg/m^2^**  Dominant/QALY | **BMI 40-50 kg/m^2^**  Dominant/QALY | **BMI 50-70 kg/m^2^**  66,744/QALY |  |
|  |  | GBP; Lifetime | **BMI 30-34.9 kg/m^2^**  20,547/QALY | **BMI ≥35kg/m^2^**  Dominant |  |  |  |
| James (2017) | Australia | Age at baseline; AGB | **Starts at age 30**  Dominant | **Starts at age 40**  Dominant | **Starts at age 50**  Dominant | **Starts at age 60**  Dominant |  |
|  |  | Age at baseline; RYGB | Dominant | Dominant | Dominant | Dominant |  |
|  |  | Age at baseline; SG | Dominant | Dominant | Dominant | Dominant |  |
| Lucchese (2017) | Italy | 10-year time horizon | **BMI 33kg/m^2^**  Male: Dominant; Female: Dominant | **BMI 37 kg/m^2^**  Male: Dominant; Female: Dominant | **BMI 42 kg/m^2^**  Male: Dominant; Female: Dominant | **BMI 52 kg/m^2^**  Male: Dominant; Female: Dominant |  |
| Klarenbach (2010) | Canada | Multiple time horizons | 9,967/QALY (10 years) | 3,257/QALY (20 years) | Dominant (Lifetime) |  |  |
| McEwen | United States | Multiple time horizons | 59,617 (2 years) | 10,651/QALY (Lifetime) |  |  |  |
| Picot (2012) | UK | Multiple time horizons | 30,388/QALY  (2 years) | 7,490/QALY (5 years) | 2,462/QALY (20 years) |  |  |
| Sanchez-Santos (2017) | Spain | 10-year time horizon | **BMI 33kg/m^2^**  Male: 5,725;  Female: 6,058 | **BMI 37 kg/m^2^**  Male: 5,430;  Female: 5,552 | **BMI 42 kg/m^2^**  Male: 3,816;  Female: 3,858 | **BMI 52 kg/m^2^**  Male: 2,786;  Female: 2,859 |  |

**Key:** AGB – adjustable gastric band; BMI – body mass index; GB – gastric band; GBP - gastric bypass (generally RYGB); LAGB – laparoscopic adjustable gastric band; LRYGB – laparoscopic Roux-en-Y gastric bypass; LSG – laparoscopic sleeve gastrectomy; NR – nor reported; ORYGB – open Roux-en-Y gastric bypass; QALY – quality-adjusted life year; RYGB - Roux-en-Y gastric bypass; SG – sleeve gastrectomy; T2D – type 2 diabetes.

## **Supplementary Information 4: Transferability assessment**

**Fig. S1: Transferability of model-based economic evaluations to the Irish context using the ISPOR questionnaire.**


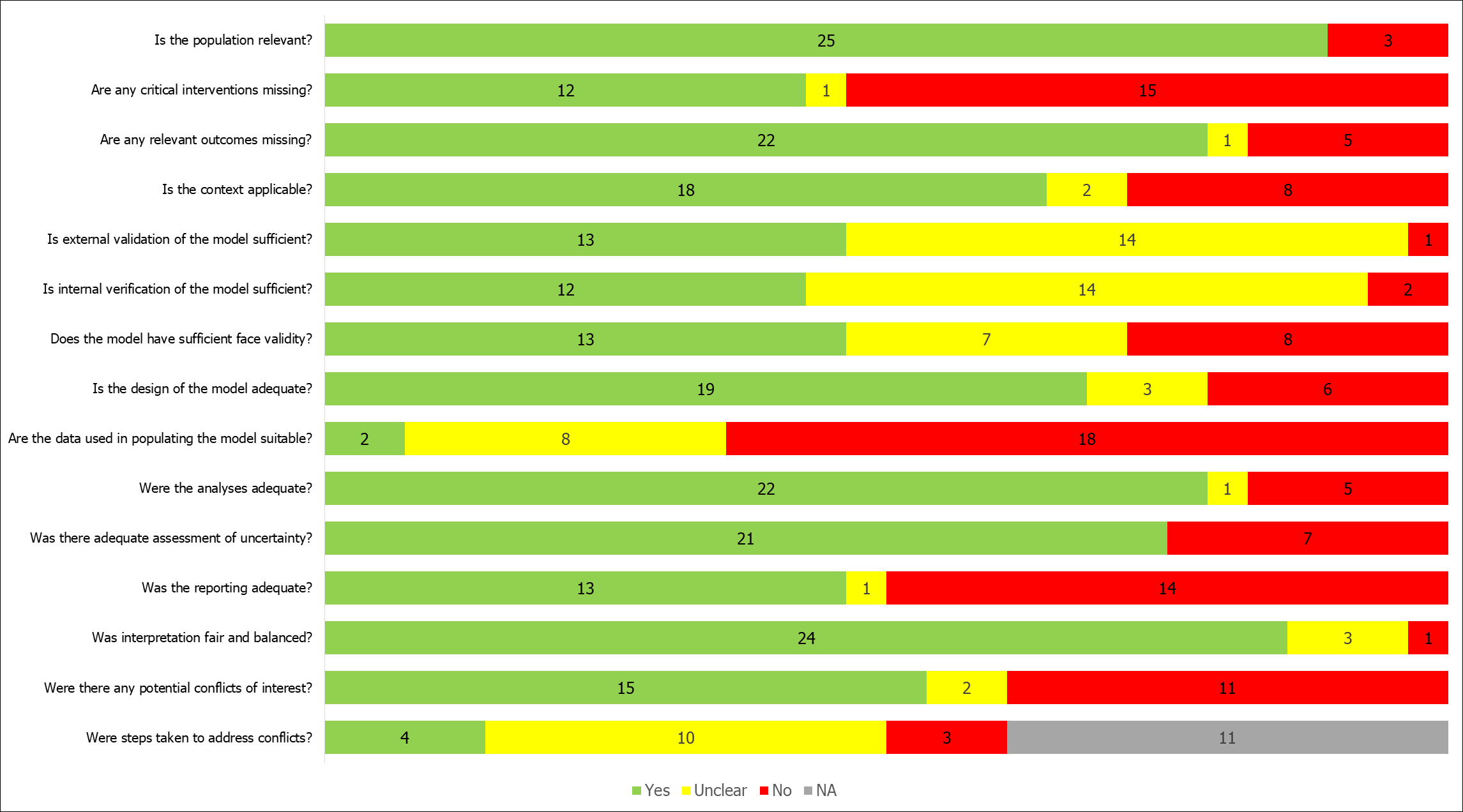


**Key:** ISPOR - International Society for Pharmacoeconomics and Outcomes Research; NA- not applicable.

**References**

1. Health Information and Quality Authority. Guidelines for the Economic Evaluation of Health Technologies in Ireland 2020 [Available from: <https://www.hiqa.ie/reports-and-publications/health-technology-assessment/guidelines-economic-evaluation-health>.

2. Iino H, Hashiguchi M, Hori S. Estimating the range of incremental cost-effectiveness thresholds for healthcare based on willingness to pay and GDP per capita: A systematic review. PLOS ONE. 2022;17(4):e0266934.

3. Anselmino M, Bammer T, Fernandez Cebrian JM, Daoud F, Romagnoli G, Torres A. Cost-effectiveness and budget impact of obesity surgery in patients with type 2 diabetes in three European countries(II). Obesity surgery. 2009;19(11):1542-9.

4. Ikramuddin S, Klingman D, Swan T, Minshall ME. Cost-effectiveness of Roux-en-Y gastric bypass in type 2 diabetes patients. The American journal of managed care. 2009;15(9):607-15.

5. Tu Y, Wang L, Wei L, Xu Y, Han X, Han J, et al. Cost-Utility of Laparoscopic Roux-en-Y Gastric Bypass in Chinese Patients with Type 2 Diabetes and Obesity with a BMI ≥ 27.5 kg/m2: a Multi-Center Study with a 4-Year Follow-Up of Surgical Cohort. Obesity Surgery. 2019;29(12):3978-86.
